# Evidence for rangewide panmixia despite multiple barriers to dispersal in a marine mussel

**DOI:** 10.1038/s41598-017-10753-9

**Published:** 2017-08-31

**Authors:** Carla R. Lourenço, Katy R. Nicastro, Christopher D. McQuaid, Rosa M. Chefaoui, Jorge Assis, Mohammed Z. Taleb, Gerardo I. Zardi

**Affiliations:** 1 CCMAR-CIMAR – Associated Laboratory, University of Algarve, Campus de Gambelas, Faro, 8005–139 Portugal; 2 Department of Zoology and Entomology, Rhodes University, Grahamstown, 6140 South Africa; 3 Department of Biology, Faculty of Natural and Life Sciences, University of Oran Ahmed Ben Bella, 31000 Oran, Algeria

## Abstract

Oceanographic features shape the distributional and genetic patterns of marine species by interrupting or promoting connections among populations. Although general patterns commonly arise, distributional ranges and genetic structure are species-specific and do not always comply with the expected trends. By applying a multimarker genetic approach combined with Lagrangian particle simulations (LPS) we tested the hypothesis that oceanographic features along northeastern Atlantic and Mediterranean shores influence dispersal potential and genetic structure of the intertidal mussel *Perna perna*. Additionally, by performing environmental niche modelling we assessed the potential and realized niche of *P*. *perna* along its entire native distributional range and the environmental factors that best explain its realized distribution. *Perna perna* showed evidence of panmixia across >4,000 km despite several oceanographic breaking points detected by LPS. This is probably the result of a combination of life history traits, continuous habitat availability and stepping-stone dynamics. Moreover, the niche modelling framework depicted minimum sea surface temperatures (SST) as the major factor shaping *P*. *perna* distributional range limits along its native areas. Forthcoming warming SST is expected to further change these limits and allow the species to expand its range polewards though this may be accompanied by retreat from warmer areas.

## Introduction

The physical environment influences species distribution patterns and shapes the genetic structure of their populations^1–4^. In the marine realm, species’ distributional arrangements and genetic discontinuities are often caused by dispersal barriers (e.g. upwelling, currents) and environmental gradients (e.g. temperature, salinity) that interrupt demographic connectivity among populations^2, 4–6^. Importantly, there is increasing modelling and experimental evidence that pronounced alterations to oceanographic features due to climatic change are re-arranging species’ genetic patterns and distributions globally^7, 8^.

Species inhabiting a specific bioregion do not all necessarily show the same genetic breaks as some are able to sustain high levels of gene flow among populations regardless of the presence of oceanographic barriers^9–11^. The absence of genetic structure has been related, for instance, to species life history traits, such as the presence of a pelagic phase and larval behaviour^9–11^. Historical events are also key drivers of genetic patterns^12^. For example, there is ample evidence that, during the Last Glacial Maximum (LGM), species retreated to restricted glacial refugia areas, persisting throughout unsuitable conditions to reveal contemporary genetic signatures that are the result of accumulated genetic diversity^13, 14^.

The Mediterranean Sea and the northeastern Atlantic are ideal regions to study the effects of dispersal barriers and environmental gradients on species distribution and genetic patterns. In the Mediterranean basin, the Strait of Sicily connects the Western and the Eastern Mediterranean regions^15^, represents a geographical break for several species^16, 17^ and is a driver of genetic differentiation^18^. The Almeria-Oran Front (AOF, stretching from Almeria, Spain to Oran, Algeria) separates the Western Mediterranean region from the Alboran Sea (Atlantic-Mediterranean) waters^19^, affecting the genetic structure of several species inhabiting both sides of the front^20^. Towards the Atlantic, the Strait of Gibraltar is the meeting point where Atlantic water enters the Mediterranean Sea at the surface, overriding the denser Mediterranean water mass^19^ and forming the focus of several studies of the effect of regional oceanographic barriers on genetic structure (reviewed in ref. 20). Along the Atlantic coast of Morocco, upwelling off Cape Ghir has been proposed as a hydrographic barrier that separates fish stocks (*Sardina pilchardus* ^21^) and shapes the genetic structure of intertidal organisms (e. g. *Mytilus galloprovincialis* ^22^; *Bifurcaria bifurcata* ^14^). Likewise, a genetic break has been detected close to Cape Boujdour in two fish ecotypes (*Engraulis encrasicolus* ^23^). Other upwelling areas or capes along this stretch of coast such as upwelling off Cape Juby and upwelling off Cape Blanc^24^ may potentially affect species’ population dynamics. Additionally, the Mediterranean and northeastern Atlantic coasts have also seen contractions and expansions of warm- and cold-water species, particularly along Portuguese shores^25, 26^, as a response to recent increases in SST (up to 0.4 °C/decade^27^). For example, two species of the brown algal genus *Fucus* (*F*. *vesiculosus* and *F*. *guiryi*) have exhibited major distributional contractions along Atlantic and Mediterranean Iberian and northern African shores linked to rates of SST warming over the last three decades^3, 7, 28^.

Recently a northward expansion of the intertidal Brown mussel *Perna perna* was described from north Africa to southern Iberia^29^. This dominant habitat-forming species occurs naturally along the northern, eastern and western coast of Africa and in the Arabian Peninsula. This intertidal subtropical species has also become invasive in the Gulf of Mexico and eastern South America (reviewed in ref. 30).

Here, we combine extensive field surveys, multimarker genetic analyses, dispersal simulations and environmental niche modelling to investigate the factors dictating the distribution and the drivers of genetic structure on *P*. *perna* along northeastern Atlantic and Mediterranean shores. Specifically we (I) use mitochondrial and nuclear markers coupled with Lagrangian particle simulations to test the hypothesis that potential dispersal and genetic structure are strongly influenced by oceanographic features (e.g. dominant currents and upwelling systems), (II) perform environmental niche modelling along the entire species’ native range to assess its potential and realized niche and predict the climatic variables of interest across the northeastern Atlantic and Mediterranean shores. Finally, the distribution of *P*. *perna* along South African shores was used as an ideal case study to highlight the relative significance of strongly correlated environmental variables. The South African coastline covers a wide range of very distinct climatic and oceanic conditions that can be divided into three major biogeographic regions^31^. These are the subtropical East Coast, the warm-temperate South Coast and the cool-temperate West Coast. Interestingly, *P*. *perna* dominates intertidal shores in the sub-tropical and warm-temperate bioregions but it is absent from the cold waters of the Benguela system^32, 33^. This distributional, oceanographic and climatic setting provides unique conditions to disentangle the relative roles played by correlated variables.

## Results

### Distribution of *Perna perna* along the Atlantic and Mediterranean Iberian Peninsula

**Figure 1:**
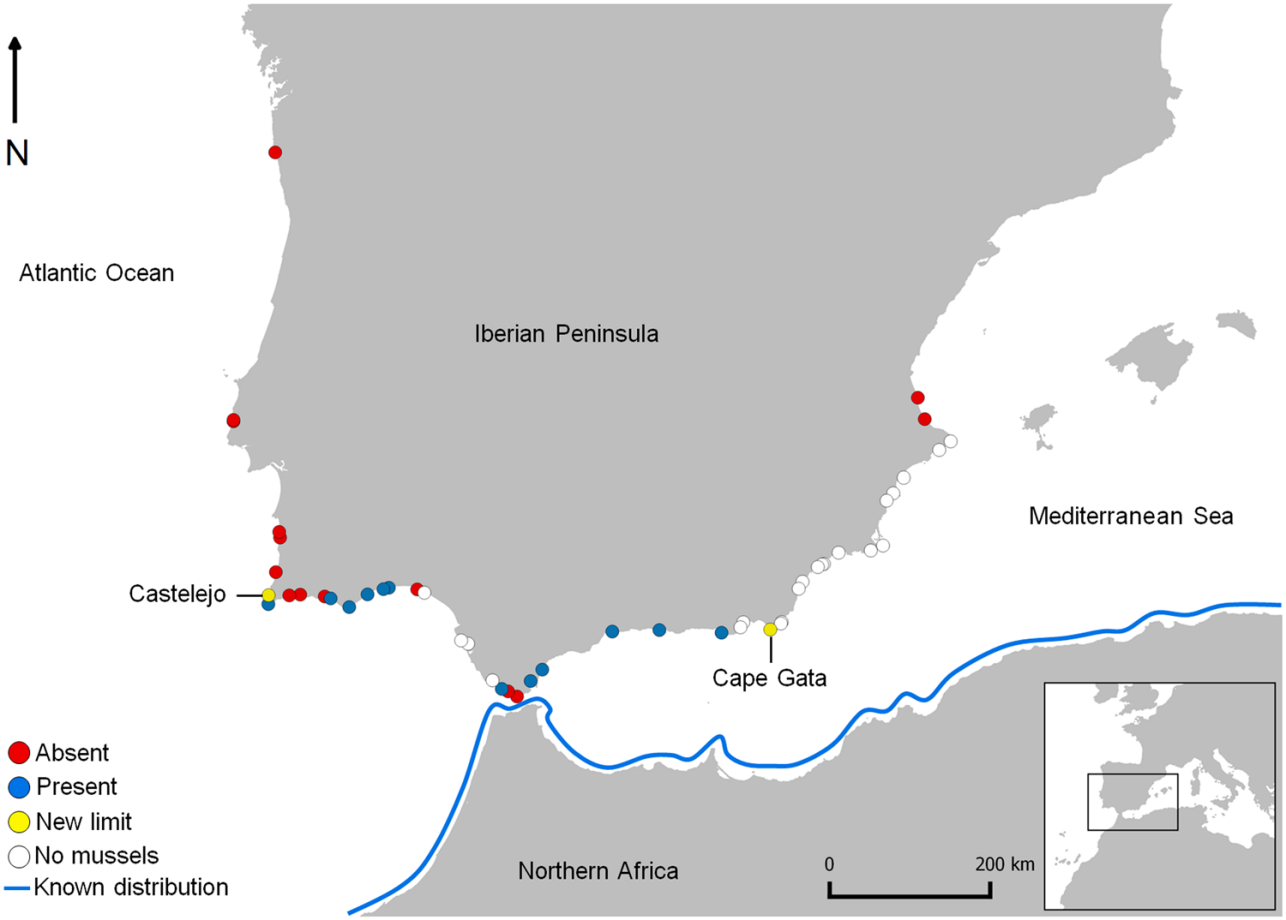
*Perna perna* range expansion along the Iberian Peninsula. Presence and absence of *P*. *perna* are marked by blue and red dots respectively. White dots represent surveyed sites where no mussel beds were found and yellow dots represent the new range limits of the *P*. *perna* distribution. The thick blue line illustrates the previously known *P*. *perna* distribution along northern Africa. Surveyed locations are described in Supplementary Table S5, from north to south and west to east. Arrow indicates north. The map was created using the open source software QGIS 2.12.3 (http://www.qgis.org/).

*Perna perna* was detected at 14 locations out of the 49 surveyed (Fig. 1). Castelejo, southwest Iberia (Portugal), was the northwesternmost location where the Brown mussel was found. North of Castelejo, individuals of this species were not detected, although the mussel *Mytilus galloprovincialis* was still abundant, indicating the existence of suitable mussel habitat. Into the Mediterranean, both species were reported as far east as Cape Gata, the easternmost limit of the Brown mussel. After a gap along the southeast coast of Iberia where intertidal mussels were entirely absent, only *M*. *galloprovincialis* reappeared.

### Genetic diversity and genetic structure of *P*. *perna* across oceanographic barriers

#### Mitochondrial DNA – Cox1

Sequences of *P*. *perna* (615 bp Cox1 gene) from 730 specimens revealed 127 haplotypes and 112 polymorphic sites (Table 1). PN and LR showed the highest number of overall haplotypes (17) while LP showed the lowest (2), likely due to the very small sample size. BZ and LR presented the highest number of unique haplotypes (8) and KR, CG and CB did not show any unique haplotypes. Whereas 94 haplotypes were private, 33 were shared among populations. Haplotype and nucleotide diversities varied between 0.481 (PM) and 1.0 (LP) and between 0.0010 (PM) and 0.0049 (LP), respectively.

**Table 1:** Genetic diversity of Cox1 gene of *P*. *perna* populations.

Population	N	H	UH	*h*	π
KR	30	8	0	0.729 ± 0.065	0.0022 ± 0.0004
BZ	32	15	8	0.821 ± 0.062	0.0030 ± 0.0005
AN	22	8	3	0.753 ± 0.069	0.0022 ± 0.0005
PN	31	17	6	0.912 ± 0.036	0.0028 ± 0.0003
CG	13	5	0	0.731 ± 0.096	0.0024 ± 0.0007
BM	31	10	5	0.714 ± 0.070	0.0022 ± 0.0006
AM	31	13	5	0.733 ± 0.085	0.0020 ± 0.0004
LA	30	14	6	0.844 ± 0.056	0.0030 ± 0.0005
TG	31	8	3	0.735 ± 0.062	0.0025 ± 0.0005
LP	2	2	1	1.000 ± 0.500	0.0049 ± 0.0024
AT	26	8	3	0.575 ± 0.113	0.0016 ± 0.0004
PM	22	7	1	0.481 ± 0.131	0.0010 ± 0.0004
TV	29	12	5	0.791 ± 0.073	0.0026 ± 0.0005
VL	29	10	4	0.687 ± 0.091	0.0022 ± 0.0005
SG	4	3	1	0.833 ± 0.222	0.0030 ± 0.0009
LR	30	17	8	0.864 ± 0.058	0.0032 ± 0.0005
RB	32	13	4	0.784 ± 0.066	0.0024 ± 0.0004
CB	30	10	0	0.699 ± 0.085	0.0021 ± 0.0004
SB	33	14	2	0.805 ± 0.064	0.0028 ± 0.0006
EB	34	15	3	0.750 ± 0.080	0.0021 ± 0.0004
ES	30	11	5	0.678 ± 0.094	0.0020 ± 0.0004
IM	29	9	4	0.648 ± 0.096	0.0013 ± 0.0003
ML	30	9	5	0.598 ± 0.103	0.0022 ± 0.0005
TT	30	12	2	0.745 ± 0.082	0.0020 ± 0.0004
BJ	29	9	2	0.613 ± 0.102	0.0013 ± 0.0003
LB	30	10	5	0.754 ± 0.063	0.0022 ± 0.0004
DK	30	8	3	0.756 ± 0.055	0.0027 ± 0.0005

N, sample size; H, number of haplotypes; UH, number of unique haplotypes; *h*, haplotype diversity (±SD); π, nucleotide diversity (±SD). Location codes as in Supplementary Table S6.

Pairwise ɸ_ST_ values were non-significant across all locations (P > 0.05; Supplementary Table S1) and ranged from 0 to 0.432; the highest estimate was found between PM and LP. High pairwise ɸ_ST_ values were recovered from populations with extremely low sampling sizes (e.g. LP, n = 2 0.039–0.432 and SG, n = 4 0.048–0.349).

Spatial analysis of shared alleles (SAShA) indicated that the observed distribution of geographical distance between pairs of haplotypes (OM) was not statistically different from the expectation (EM) under panmixia (OM = 885.30 km, EM = 892.06 km, P = 0.773).

The Akaike Information Criterion corrected for small sample sizes (AICc) selected TrN + G as the best-fit model to be implemented in Arlequin software (gama shape = 0.932). AMOVA analyses attributed most of the variation to within populations (99.52%, P = 0.126; Table 2).

**Table 2:** AMOVA from the 27 populations of *P*. *perna* distributed over seven distinct groups based on mitochondrial Cox1 gene and seven nuclear microsatellites.

Source of variation	d.f.	Sum of squares	Variance components	Percentage of variation	ɸ-statistics	P-value
**Cox1 gene**						
Among groups	6	6.327	0.0037	0.53	0.0053	0.006
Among populations within groups	20	13.717	−0.0004	−0.05	−0.0005	0.505
Within populations	703	488.654	0.6951	99.52	0.005	0.126
Total	729	508.699	0.6985			
**Microsatellites**						
Among groups	6	16.79	0.0004	0.02	0.0002	0.351
Among populations within groups	20	54.17	0.0007	0.03	0.0003	0.76
Within populations	1437	3836.05	2.6695	99.96	0.0004	0.728
Total	1463	3907.01	2.6707			

**Figure 2:**
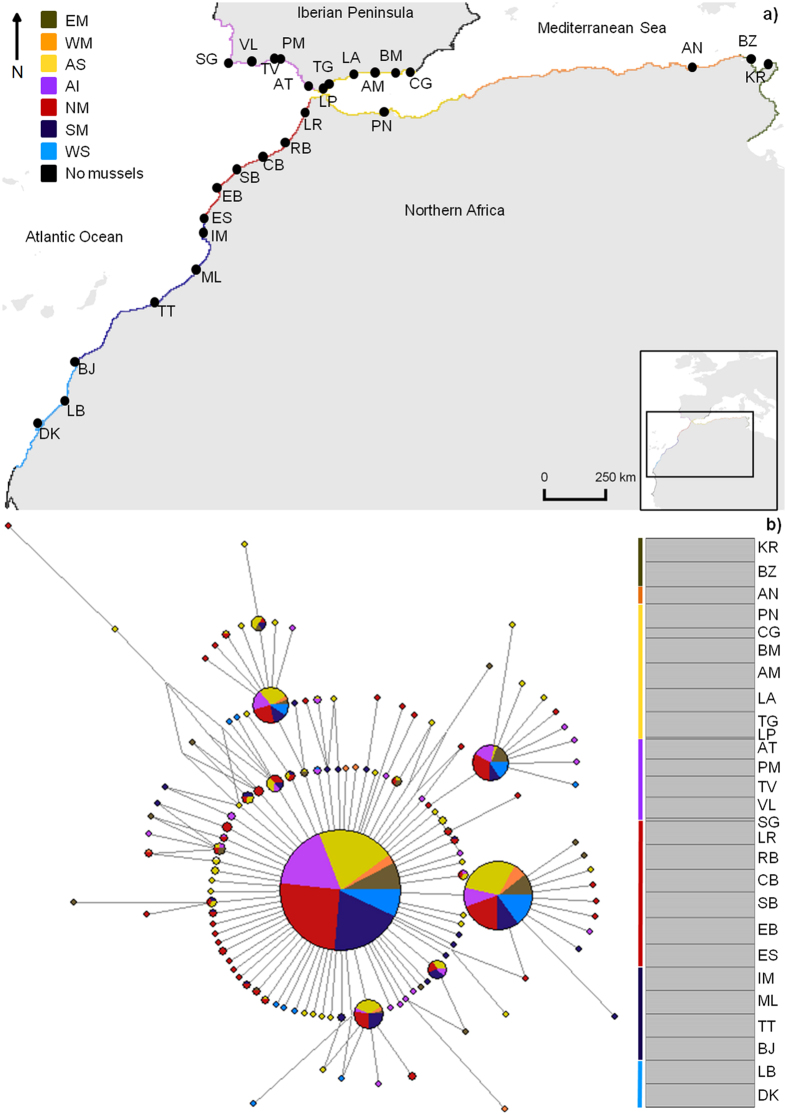
Genetic structure of *P*. *perna* across northeastern Atlantic and Mediterranean shores. (**a**) Oceanographic regions based on dispersal potential simulation of *P*. *perna*. Colours along the shore depict distinct oceanographic regions. EM, Eastern Mediterranean; WM, Western Mediterranean; AS, Alboran Sea; AI, Atlantic Iberia; NM, Northwestern Morocco; SM, Southern Morocco; WS, Western Sahara; “No mussels” represents areas where the species is either not present (Atlantic central Iberia and northwards; southeastern Iberia and northeastwards) or it was not sampled (southernmost coast of the northern African region). Black solid circles represent sampling locations as in Supplementary Table S6. The map was created using the open source software QGIS 2.12.3 (http://www.qgis.org/). (**b**) Median-joining haplotype network of Cox1 gene (left) and bayesian analysis summary plot (each bar represents one individual) obtained from STRUCTURE i.e. *K* = 1 (right). Circle size is proportional to haplotype frequency. Colours indicate the group origin of a haplotype. Grey line represents the proportion of mutational steps. Coloured bars depict expected genetic clusters.

The median-joining haplotype network reconstruction of *P*. *perna* revealed one single clade, and no genetic differentiation between *a priori* expected distinct groups (Fig. 2). The star-shaped network presented two main central haplotypes widespread at all groups. Generally, both shared and private peripheral haplotypes differed from the centre in one or two mutational steps. Haplotypes were shared irrespective of the geographic distance between groups (i.e. haplotypes shared between WM and AI or between WM and WS).

#### Microsatellite markers

Out of 732 individuals, 373 alleles were detected in seven loci. The total number of alleles per locus ranged from 12 to 134. Excluding locus P16, there was no clear evidence for large allele drop-out, stuttering or null alleles at a frequency higher than 0.2. Expected (H_E_) and observed (H_O_) heterozygosities varied between 0.536 (CB) and 0.796 (LP) and 0.643 (SG) and 0.773 (AN), respectively, resulting in a minor heterozygosity deficit (F_IS_ ranged from 0 to 0.128). Population genetic diversity standardized to the smallest sample sizes, Â_(2)_ and Â_(22)_, varied between 2.714 (LP) and 3.123 (CB) and between 11.966 (IM) and 14.714 (PM), respectively. A total of 105 unique alleles were described with CB reporting the highest number (10; Table 3).

**Table 3:** Genetic analyses of *P*. *perna* populations based on nuclear microsatellite markers.

Population	N	H_E_	H_O_	Â	Â_(2)_	Â_(22)_	F_IS_	UA
KR	30	0.776	0.737	15.714	3.061	13.483	**0**.067**	7
BZ	32	0.770	0.712	15.857	3.011	13.190	**0**.091**	4
AN	22	0.761	0.773	13.429	2.972	13.429	0.009	1
PN	31	0.754	0.743	16	2.977	13.422	0.032	7
CG	13	0.747	0.747	9.857	2.998	—	0.039	0
BM	32	0.753	0.736	17	3.004	14.012	0.039	7
AM	33	0.749	0.690	17	2.980	13.817	**0**.093**	2
LA	30	0.765	0.743	15.571	3.031	13.438	**0**.046*	4
TG	33	0.751	0.703	15.714	2.977	12.876	**0**.079**	7
LP	2	0.536	0.714	2.714	2.714	—	0	0
AT	26	0.783	0.769	15.714	3.102	14.360	0.039	2
PM	22	0.767	0.716	14.714	3.052	14.714	**0**.090**	4
TV	27	0.750	0.740	13.857	2.982	12.557	0.032	0
VL	27	0.780	0.773	15.714	3.088	14.227	0.028	1
SG	4	0.607	0.643	4.5714	2.812	—	0.085	1
LR	30	0.780	0.744	16.429	3.098	14.027	**0**.063**	4
RB	32	0.779	0.751	16	3.046	13.411	**0**.052*	4
CB	29	0.796	0.708	16.429	3.123	14.394	**0**.128**	10
SB	33	0.754	0.701	17.143	3.012	14.046	**0**.085**	3
EB	34	0.769	0.731	16.429	3.021	13.354	**0**.064**	6
ES	30	0.751	0.712	15.286	2.976	13.063	**0**.069**	6
IM	30	0.728	0.726	13.857	2.899	11.966	0.019	5
ML	30	0.757	0.763	16.429	3.014	13.965	0.008	1
TT	30	0.751	0.762	16	2.987	13.629	0.003	5
BJ	30	0.769	0.731	15.571	3.006	13.247	**0**.066*	2
LB	30	0.765	0.730	15.429	3.012	13.228	**0**.063*	6
DK	30	0.733	0.695	15.857	2.915	13.457	**0**.069*	6

N, number of individuals per population; H_E_, expected heterozygosity; H_O_, observed heterozygosity; Â, allelic richness represented by mean number of alleles per locus per population; Â_(n)_ allelic richness standardized to smallest sample sizes; F_IS_, inbreeding coefficient; UA, unique alleles. Significant values of F_IS_ are in bold. *P < 0.05; **P < 0.01. Locations codes as in Supplementary Table S6.

F_ST_ and Jost’s D ranged from 0 to 0.034 and from 0 to 0.041, respectively (Supplementary Table S2), and showed no significant differences in any pairwise comparisons (F_ST_ lower and upper 95% confidence interval limits ranged from −0.174 to −0.004 and from 0.005 to 0.209, respectively; Jost’s D lower and upper 95% confidence interval limits ranged from −0.179 to −0.007 and from 0.022 to 0.279, respectively; Supplementary Table S2).

The neighbour-joining tree based on proportion of shared alleles gave clear evidence of the absence of geographical topology (Supplementary Fig. S1).

The log probability of the data (L(K)) returned from the Bayesian admixture analyses implemented in STRUCTURE suggested K = 1 as the best fitting K (Fig. 2). Although using the ΔK method^34^ proposed K = 2 as the best fitting K, the two proposed resolved clusters were consistently present at all populations, thus excluding any geographical or genetic separation.

AMOVA indicated that most of the genetic variation arose within populations (99.96%; P = 0.728, Table 2).

Discriminant analyses of principal components (DAPC) suggested K = 9 as the best fitting number of clusters. All clusters were distributed across the entire study area, revealing no spatial differentiation among groups (Supplementary Fig. S2).

#### Simulations of dispersal potential

The Lagrangian particle simulations (LPS) using HYCOM ocean velocity fields over the 11-year period released ~3300 particles per cell (32.43e10^6^ particles in total). On average, the particles drifted for 110.4 km ± 123.9 (maximum 1019 km) and most connectivity events were produced in the first days of ocean drifting (mean transport time of 2.1 days ± 1.9).

The linear models using ocean connectivity estimates and shortest marine distances failed to explain the genetic differentiation of *P*. *perna*, and none had a better ability to explain the data, in terms of either Adjusted R-squared or Akaike criteria (Supplementary Fig. S3).

The identification of oceanographic regions performed with the leading eigenvector algorithm on the stepping-stone connectivity matrix showed a significant modularity value of 0.76 (P < 0.001). The algorithm was allowed to identify 10 distinct regions (Fig. 2), with their breaks in Cape Roca (Western Iberia, Portugal; the species is absent from this region), Strait of Gibraltar (Atlantic-Mediterranean meeting point), Cape Gata (southeastern Iberia, Almeria, Spain (Almeria-Oran Front); the species is absent eastward of this break), Oran (Mediterranean northern Africa, Algeria, Almeria-Oran Front), Strait of Sicily (northeastern Africa, Tunisia), Essaouira (northwestern Africa, Morocco), Cape Boujdour (northwestern Africa, Western Sahara) and Cape Barbas (northwestern Africa, Western Sahara; no samples acquired south of this break). These break points (i.e., oceanographic barriers) prevented most particles from connecting coastal cells between oceanographic regions during at least one dispersal event (Table 4). With the exceptions of the barriers separating AI from AS (potential to reduce connectivity: 81%), AS from WM (potential to reduce connectivity: 85%) and NM from AS (potential to reduce connectivity: 83%), all oceanographic barriers had the potential to reduce the connectivity between cells by 93% to 100%, with a general increase in reducing connectivity as the relative distance between regions increased (Table 4).

**Table 4:** Strength of oceanographic barriers given by the percentage reduction in connectivity between oceanographic regions.

		To region						
		EM	WM	AS	AI	NM	SM	WS
From region	EM	—	94	100	100	100	100	100
	WM	95	—	99	100	100	100	100
	AS	100	85	—	99	99	100	100
	AI	100	99	81	—	98	100	100
	NM	100	99	83	98	—	99	100
	SM	100	100	100	100	98	—	93
	WS	100	100	100	100	100	98	—

EM, Eastern Mediterranean; WM, Western Mediterranean; AS, Alboran Sea; AI, Atlantic Iberia; NM, Northwestern Morocco; SM, Southern Morocco; WS, Western Sahara.

### Environmental niche modelling

Pearson’s correlation test revealed strong correlations between sea surface temperature (SST) and surface air temperature (SAT) and between nitrate and phosphate concentrations. Although the discarding of correlated variables may be an arbitrary procedure^35^, the most ecologically representative variables for intertidal species were prioritized in each correlation (e.g. ref. 36), i.e. SST and nitrate concentration. After Pearson’s correlation test minimum and maximum SST, nitrate concentration, salinity, cloud cover and the significant wave height were selected to perform the analyses.

**Figure 3:**
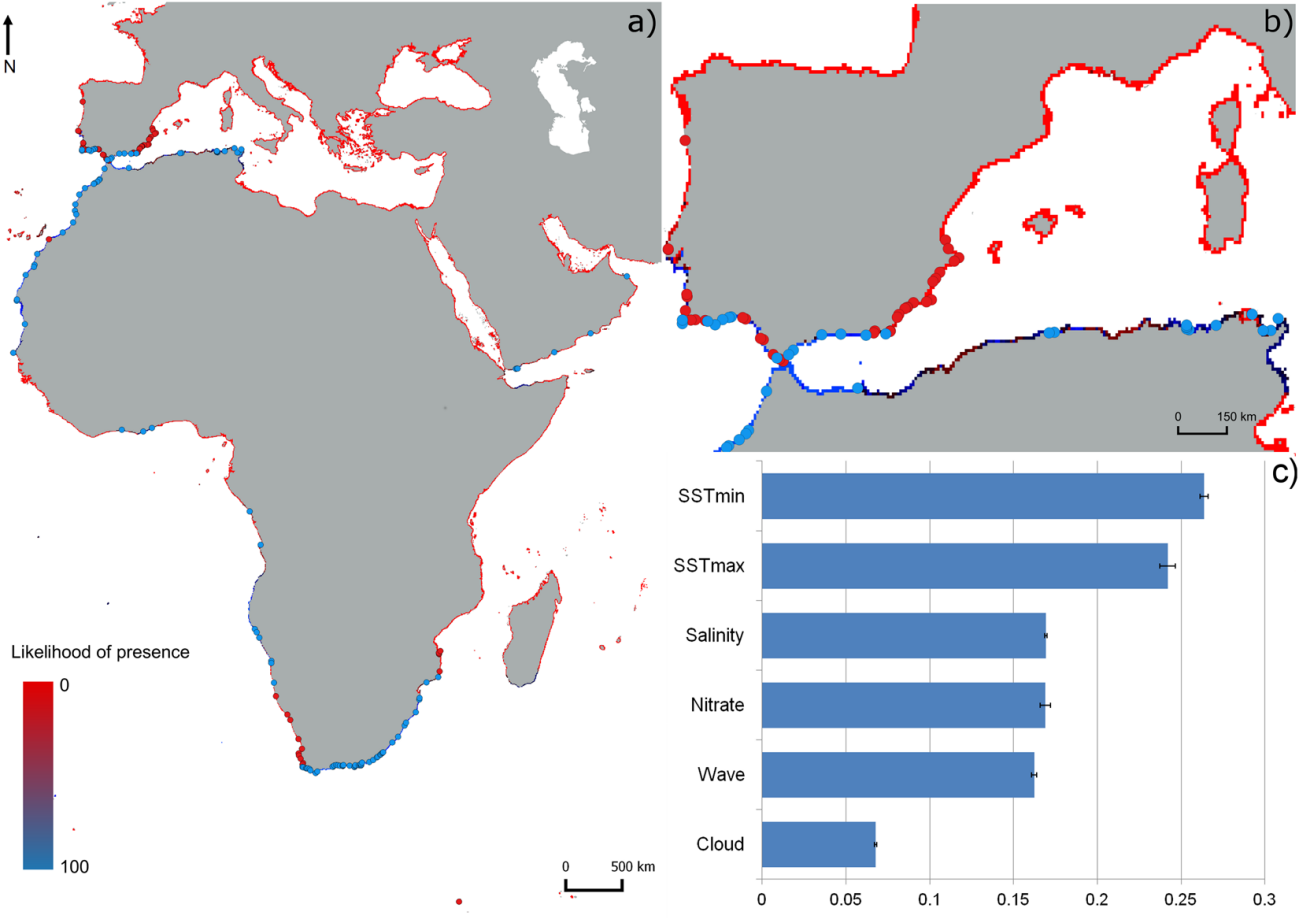
Predicted native distribution for the brown mussel *P*. *perna* derived by averaging an ensemble of presence-absence algorithms. (**a**) overall distribution, (**b**) *P*. *perna* distribution along the expanding front in the Northern Hemisphere, (**c**) Mean scores of the relative importance of the environmental variables obtained from the ensemble. Blue and red dots represent presence and absence data, respectively, obtained from field surveys and records in the literature (see Supplementary Table S7). The map was created using the open source software QGIS 2.12.3 (http://www.qgis.org/).

The ensemble produced with the best models (TSS > 0.7) resulted in an accurate overall description of *P*. *perna* native distribution, including its expanding front towards southern Iberia (Fig. 3). Along northern Africa, the niche model predicted a distribution from central Senegal north into the Mediterranean, as far as central-eastern Tunisia (Fig. 3). In addition, the prediction indicated that suitable habitat could potentially be found from southeastern Spain to central Portugal. While the probability of *P*. *perna* being present on Mediterranean Spanish shores was high, towards the Atlantic the predicted likelihood decreased. On southwestern Iberian shores, the species is absent from several locations where it could be expected. Surprisingly, short portions of potentially suitable habitat were detected along the warm equatorial African coast (in Ghana and Ivory Coast) and the Arabian Peninsula (Yemen and Oman) under the effect of upwelling cells. RandomForest (RF) performed better than other techniques (AUC = 0.939 ± 0.018 and TSS = 0.775 ± 0.044, Supplementary Table S3). The evaluation of the ensemble produced the following ROC-derived scores: AUC = 0.968, sensitivity = 99.099, and specificity = 88.928; TSS = 0.879, sensitivity = 99.099, specificity = 88.839. By obtaining the highest score (0.26), minimum SST was the predictor that best explained the distribution of *P*. *perna* (Fig. 3), when modelled alone in comparison with other predictors.

#### Case study: South Africa

**Figure 4:**
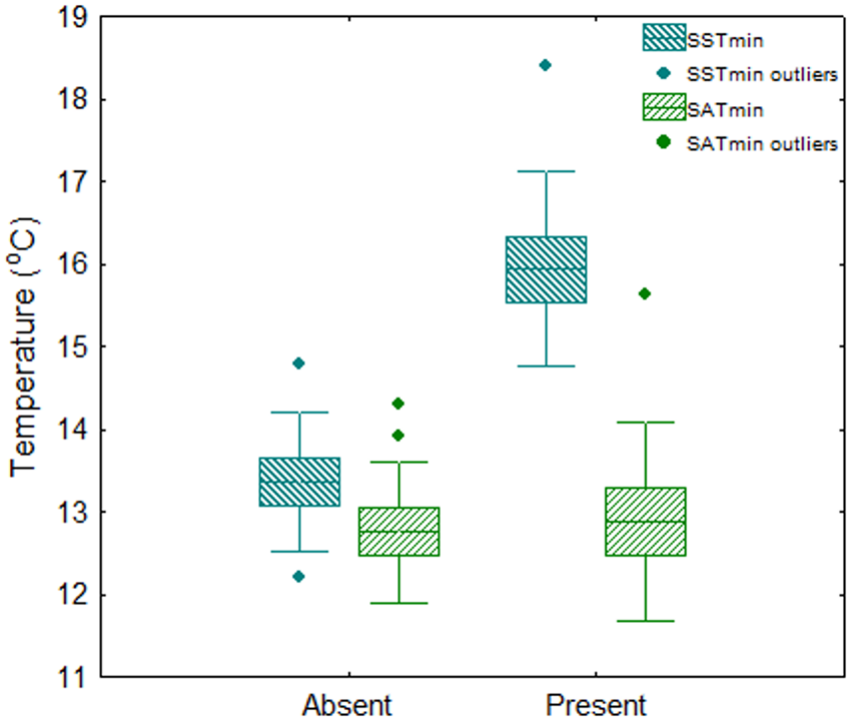
Box-plot of minimum sea surface temperature (SST) and surface air temperature (SAT) of the two South African regions where *P*. *perna* is absent (cold water) or present (warm water). Box-plot depicts the mean (horizontal line), the standard error (bottom and top of the box) and the standard deviation (whiskers).

Minimum SST was significantly lower where *P*. *perna* is absent compared to where the species is present (all trials P < 0.001, Fig. 4, Supplementary Table S4). In contrast, minimum SAT did not show any significant difference between the two groups of locations (trial 1 P = 0.796; trial 2 P = 0.511; Trial 3 P = 0.063; Supplementary Table S4). Although only one subset is shown, the results were consistent for all three.

## Discussion

Lagragian particle simulations (LPS) performed along northeastern Atlantic and Mediterranean shores found evidence of the existence of several oceanic barriers with the potential to reduce connectivity between populations to a high degree, as previously described for several marine species. However, our results indicate a lack of genetic differentiation of *Perna perna* populations across the study area. Moreover, the niche modelling framework highlights the key role of minimum sea surface temperature (SST) in shaping *P*. *perna* distributional range limits along its native areas including the southern Iberian expanding front.

### Lack of genetic structure in face of multiple oceanographic barriers

In marine organisms with sessile or highly sedentary adults, genetic structuring is often directly linked to dispersal barriers influencing the transport of planktonic propagules^32, 37^, though barriers for dispersal do not affect all species equally^10, 11^.

The general assumption is that an extended dispersal phase coupled with very large population sizes will result in high levels of connectivity over large geographic scales and across biogeographic provinces^38, 39^. However, in recent years, several studies have challenged this view, showing significant genetic structure in organisms with long pelagic larval stages^40, 41^. For example, South African *P*. *perna* populations are characterised by marked genetic heterogeneity at both meso***-*** and macro***-*** spatial scales. At large scales, strong genetic division between two geographically defined groups of populations (temperate vs subtropical/tropical) in southern Africa are highlighted by mitochondrial (Cox1)^32^ and nuclear (ITS^30^; microsatellites^42^) markers, though this appears to reflect the evolutionary history of the species. At smaller scales, mitochondrial data show that populations within the temperate group that occupy different bays are genetically distinct from each other and from populations on the open coast^43^.

Despite numerous potential oceanographic barriers, selective environmental gradients and in contrast to marked genetic structure described for various species, our multilocus approach unequivocally pointed to a northeastern Atlantic and Mediterranean panmitic population of *P*. *perna*. Lagrangian particle simulations (LPS) detected 10 oceanographic regions with several breaking points. This result supports previous studies reporting oceanographic barriers and genetic discontinuities at (I) the Strait of Gibraltar, meeting point between Atlantic and Mediterranean (Alboran Sea) waters^20, 44^; (II) the Almeria-Oran front, separating the Alboran Sea from Western (European and African) Mediterranean^20^; (III) the Strait of Sicily, connecting Western and Eastern Mediterranean^18^, (IV) Cape Ghir upwelling^14, 22^ and around (V) Cape Boujdour. However, these known oceanographic barriers did not cause genetic discontinuities in *P*. *perna*. Additionally, the LPS detected two possible new breaking points: (VI) Cape Roca, central Portugal and (VII) Cape Barbas, Western Sahara. The Atlantic Iberian coast has been the focus of several studies investigating the genetic structure of marine species^45, 46^. Despite clear genetic discontinuities along the west coast of Portugal^3, 28, 47^, the potential of Cape Roca to act as an oceanographic barrier to species distribution and genetic structure warrants further investigation. Interestingly, this break point matches the expected northernmost limit for *P*. *perna* distribution in Iberia, detected through the niche modelling approach (Fig. 3; see discussion below).

Lastly, Cape Barbas is known to separate two upwelling cells in the Western Sahara (Cape Boujdour to Cape Barbas^48^ and Cape Barbas to Cap Blanc^49^) and for having the potential to delimit the distribution of marine species (*Sardina pilchardus*)^50^. Although LPS predicted an oceanographic barrier for *P*. *perna* larval dispersal at Cape Barbas, future studies are needed to understand if this translates into a genetic discontinuity. Studies along this region are scarce, presumably due to continuous conflict; the few genetic studies performed rarely target sites south of the Dakhla Peninsula (but see ref. 51), and lack the resolution to investigate the effect of Cape Barbas in shaping genetic structure of marine species.

The oceanographic barriers identified by LPS have the potential to reduce connectivity of coastal cells by more than 80% between distinct oceanographic regions. Thus, the unexpected lack of genetic structure in *P*. *perna* across such strong oceanographic barriers most likely results from a combination of species’ life history traits, habitat continuity and stepping stone movements among regions. *Perna perna* is a broadcast spawner with a pelagic phase of 2–3 weeks^52^, a period that might be extended as a result of larval and postlarval behaviour^53, 54^, and its distribution across northern Africa extends continuously through thousands of kilometres, from Tunisia to Senegal.

Habitat continuity and stepping-stone dynamics influence species genetic structure^18, 55^. The great dispersal capacity of *P*. *perna* through its long planktonic phase, combined with the existence of extensive areas of continuous habitat, is likely to account for the lack of genetic structure, by enabling individuals to connect locations hundreds of kilometres apart through consecutive stepping-stone movement steps and consequently mixing gene pools. Despite the strength of oceanographic barriers, the simulations pointed to some degree of putative connectivity between adjacent regions (potential to reduce connectivity from 81% up to 99%), increasing the potential of *P*. *perna* to colonize suitable habitats on a year-to-year stepping stone scenario. Additionally, hydrological features such as oceanic currents may enhance or limit large-scale genetic homogenization of a species’ gene pool^56, 57^. The slope currents contouring northern Africa and southern Iberia^58, 59^ may play a role in enhancing *P*. *perna* dispersal by promoting larval transportation and homogenizing the gene pool across the study area, although the general patterns of the dispersal simulation suggest otherwise. Nonetheless, these were the regions of our study where the detected oceanographic break points allow higher potential for connectivity (reductions down to 81%), potentially contributing to the lack of genetic structure described in this study.

Evidence of panmixia has been previously provided for other species across some of the regions considered in this study (e. g. mitochondrial marker (D loop): fish species *Diplodus sargus*, *Pagellus bogaraveo*, *Pagrus pagrus* and *Scomber japonicus* ^20^; mitochondrial markers (Cox1 and 16S): abalone *Haliotis tuberculata*, chiton *Chiton olivaceus* ^60^). While we explain the genetic pattern of *P*. *perna* in terms of life history traits and ecological variables, other panmitic genetic signatures have been explained as unlikely to be due to dispersal, but rather the result of fluctuations in populations size (i.e. extinction/colonization and migration) and historical demography^20, 60^.

Despite general evidence of panmixia across several taxa^20, 60^, rangewide panmixia is extremely rare^9, 61^. The panmitic genetic pattern described from northern Africa and southern Iberia strongly contrasts with the genetic pattern observed from South African populations of *P*. *perna*. In South Africa, the Brown mussel shows a pronounced genetic break which corresponds to a marked biogeographic disjunction between the temperate and subtropical provinces, separating two distinct genetic lineages^32^. Although *P*. *perna* shows similar life history traits, habitat continuity and stepping stone dynamics in both northern and southern Africa, the genetic structure in South Africa has a historical origin and is presently maintained by the nearshore influence of the powerful Agulhas current, which impedes larval dispersal and thus promoting local adaptation^2, 32, 42^. Along northern Africa and southern Iberia, neither historical nor contemporaneous genetic discontinuities were detected, as evidenced by the complete agreement between markers (mitochondrial and nuclear markers) with different rates of evolution.

### Minimum SST explains *P*. *perna* northern native range limits

The Iberian Peninsula is an interface region where several cold- and warm-water species reach their southern or northern distributional limits^25, 26^, with several new distributional patterns resulting from range expansions and contractions being attributed to warming SST (e.g. the phaeophyte *Fucus vesiculosus* ^3^; the pulmonate limpet *Siphonaria pectinata* ^62^, but see ref. 63). The Brown mussel, *P*. *perna*, has been intermittently recorded from Portuguese shores^29, 64^ from fossil records, kitchen middens and museum specimens dating from the Ancient Neolithic (8000–5000 BP), the Medieval Warm Period (XII century), the late XIX (1888–1899) and the early XX (1938) centuries^64^. These occurrences coincided with warmer periods of SST (see refs 64–66) and mirror a close relationship between the successive Iberian colonisation events of *P*. *perna* and major warmings of SST^64^. Given how rising SST has shaped the distribution of *P*. *perna* both now and in the past, we can expect further poleward colonisation as warming continues^67^. Such distributional changes have already been demonstrated for other intertidal species across the study region. For example, the pulmonate limpet *S*. *pectinata* has expanded its distribution 185 km northwards since 1940, presumably driven by an increase in SST^62^). Similarly, new northern limits for the limpet *Patella rustica* ^68^ and macroalgae such as *Codium adhaerens* and *Padina pavonica* on Atlantic Iberian shores have been related to warming of SST^26^.

Seawater temperature, whether minimum, mean, maximum SST or water bottom temperature, is a key variable in explaining the modelled distribution of nearshore species. In some case studies, SST is found to be the main environmental contributor in the projection of species distribution (e.g. gastropod species *Littorina saxatilis*, the crab *Carcinus maenas* and the tunicate species *Styela clava* in ref. 69). Even when not the most significant contributor, water temperature still remains one of the top environmental predictors explaining nearshore species distribution^6, 35, 70–72^. For example, out of 24 variables, SST is among the top range distribution predictors of two intertidal gastropod cryptic species of the genus *Melampus* ^70^. Moreover, bottom water temperature and depth had the greatest impact on the distribution of 14 benthic species out of 10 environmental variables modelled in^71^. The results of the present study further support the determinant role of SST in explaining the distribution of nearshore organisms. Minimum SST was the predictor that best explained *P*. *perna* native distribution. The sharp drop in SST at Cape Gata^19, 73^ sets the northeastern limit of *P*. *perna* Iberian distribution, highlights the lack of suitable conditions farther east and is consistent with a preference for subtropical conditions. As SST and surface air temperature (SAT) are commonly linked to latitudinal gradients, difficulties arise when trying to tease apart their separate effects on biogeographic patterns^74^. Although SST and SAT were positively correlated across the entire native distribution, additional analyses of thermal regimes in South Africa provide an ideal scenario to disentangle the relative significance of SAT and SST. South African shores are characterised by sharp environmental clines associated with abrupt species distributional changes^31^. *Perna perna* is absent from the cool west temperate province (minimum SST ranging 12.2–14.8 °C, Fig. 4; winter SST 13–15 °C^75^) but distributed across the southern warm temperate one (minimum SST ranging 14.5–18.7 °C, Fig. 4; winter SST 15–19 °C^75^). The west coast of South Africa is permanently affected by the cold waters of the Benguela upwelling system, whereas the south flowing Agulhas current transports warm water along the east and south coasts of South Africa^76^, offering suitable conditions for *P*. *perna*. Our results show that minimum SST are significantly lower along the west than the south coast, while SAT shows no such pattern. Our findings support previous studies indicating SST as a major predictor of intertidal species distribution, increase our understanding on the role of minimum SST and SAT in setting species range limits at a regional level and emphasise the importance of low SST in limiting *P*. *perna* distribution in the Iberian Peninsula. The inability of *P*. *perna* to persist under cold water conditions can be the result of sub lethal effects on its metabolism. Extremely low densities of rare adult *P. perna* individuals along the west coast of South Africa indicate that the species can survive after settlement but it is unable to reproduce under low SST, even where food availability is particularly high^33^.

Total agreement between modelled and observed distributions reflects a complete occupation of the species potential niche^6^. This has been shown for the invasive distribution of the blue mussel *Mytilus galloprovincialis* along the shores of South Africa^6^. *Mytilus galloprovincialis* arrived on South African shores in the late 1970s^77^ and has now occupied all suitable habitats which correspond to about 2,800 km of coast^6^. In the present study, an extremely high, nearly complete agreement between observed and modelled niche ranges was obtained (sensitivity = 99.099), although slightly lower than the previous example. The slight disagreement between observed and predicted niche ranges implies that some suitable habitats still remain unoccupied and that additional abiotic or biotic factors may affect the distribution of *P*. *perna*. The northwesternmost observed Iberian distributional limit of *P*. *perna*, Castelejo (Portugal), is approximately 180 km south of the predicted limit (Cape Roca), depicting a stretch of coast that has not yet been colonised, despite the existence of suitable environmental conditions. Plausible explanations for this discrepancy include insufficient time for colonisation or biological interaction. *Perna perna* specimens could be absent from most southwest Portuguese shores due to a slow occupation of the potential niche. However, the species is characterized by highly invasive behaviour, spreading for hundreds of km under suitable environmental conditions^78, 79^. *Perna perna*, could, however, be ecologically excluded from suitable habitat. Interaction with recipient communities is a major determinant of the potential establishment of a species^80^. As *Mytilus galloprovincialis* exhibits competitive dominance over *P*. *perna* on South African shores (at the upper intertidal)^81^, the Brown mussel might be similarly outcompeted by *M*. *galloprovincialis* from southwest Portugal by means of higher recruitment rate^82^, faster growth^83^ and/or greater colonisation ability^84^.

## Conclusions

We report the most extensive genetic continuity so far observed for an intertidal organism distributed across northeastern Atlantic and Mediterranean shores, despite the existence of several oceanographic barriers to dispersal that are predicted by Lagrangian particle simulations and previously described in the literature. The results highlight the importance of adopting a multidisciplinary approach based on species distribution, larval dispersal simulations, genetic characterization and ecological niche modelling if one wishes to understand how species distributions and range limits are likely to respond to climate warming. We also emphasise that this approach identifies potential distributions; realised ranges will reflect the additional effects of biological interactions.

## Methods

### Distribution of *Perna perna* along the Atlantic and Mediterranean Iberian Peninsula

The distribution of *P*. *perna* along the Atlantic and Mediterranean Iberian Peninsula (from the northwestern Portuguese Atlantic coast, Viana do Castelo 41°41′57.85″N; 08°51′23.81″W, to the Mediterranean Spanish coastline, Cullera 39°11′16.26″N; 00°13′17.20″W; Supplementary Table S5) was investigated through extensive field surveys during low spring tides between November 2011 and July 2016 at 49 natural or manmade (e.g., pontoons, pilings and seawalls) intertidal habitats. At each location, two observers assessed presence or absence of *P*. *perna* by performing approximately 60 min searches across all microhabitats. Because *Mytilus galloprovincialis* is the dominant intertidal mussel species of these shores and is known to co-exist with *P*. *perna* in temperate regions^32, 85^, its conspicuous presence was considered an indication of suitable habitat for *P*. *perna*.

### Genetic diversity, genetic structure and dispersal potential of *P*. *perna* across oceanographic barriers

*Perna perna* individuals (sample sizes in Table 1) were collected between November 2011 and March 2014 from 27 locations (Supplementary Table S6). Mantle tissue (20–30 mg) was dissected from each individual, preserved in 96% ethanol and stored at −20 °C. Total genomic DNA extraction was performed using a standard Proteinase K protocol adapted from ref. 86. The primers LCOI 1490, 5′-GGT CAA CAA ATC ATA AAG ATA TTG G-3′ and HCO 2198, 5′-TAA ACT TCA GGG TGA CCA AAA AAT CA-3′^87^ were used for polymerase chain reaction (PCR) amplification of Cox1 region. PCR amplification was performed in a 25 μl reaction volume containing 10 to 100 ng of total DNA, 0.2 μM of each primer, 0.08 mM of each dNTP, 2 mM of MgCl_2_, 1x GoTaq Flexi Buffer (Promega, USA) and 1 U GoTaq DNA Polymerase (Promega, USA). Amplification used an initial denaturation during 2 min at 94 °C followed by 35 cycles of denaturation at 94 °C for 60 s, annealing at 55 °C for 60 s, extension at 72 °C for 90 s and a final extension at 72 °C for 5 min. PCR products were then purified for sequencing using ExoSap (USB Co., USA) and sequenced directly with PCR primers using the BigDye Terminators version 3.1 Cycle Sequencing Kit (Applied Biosystems, Foster-City, CA) in an ABI PRISM 3130 genetic analyzer (Applied Biosystems). Eight microsatellite loci P01, P02, P05, P08, P16, P20, P26 and P27 were amplified and genotyped as in ref. 88.

#### Genetic analyses

We applied a multimarker approach to increase the power to detect genetic discontinuities^89^. Additionally, by combining mitochondrial (slower mutation rate) and nuclear (higher mutation rate) markers, we aimed to understand whether historical and/or contemporary oceanographic barriers are responsible for restricting the gene flow in *P*. *perna* (see refs 89 and 90).

Mitochondrial DNA: DNA sequences were edited and aligned using Geneious 4.8.2 (Biomatters Ltd.). DnaSP 5.0^91^ was used to evaluate haplotype (*h*) and nucleotide (π)^92^ diversities for individuals collected at the same location (hereafter referred to as a population). Total numbers of haplotypes (H) and unique haplotypes (UH) were estimated for each population in DNAcollapser from FaBox^93^.

The Akaike Information Criterion corrected for small sample sizes (AICc) was used in jModelTest 0.1.1^94^ to select the best fitting model of sequence evolution to analyse the dataset in Arlequin 3.11^95^. Genetic differentiation between pairs of populations was calculated by estimating ɸ_ST_ based on haplotype frequency. Statistical significance was assessed by performing 10,100 permutations under the null hypothesis of no differentiation, and adjusted with q-value correction^96^ (implemented in R^97^). Spatial analysis of shared alleles (SAShA)^98^ was used to complement ɸ_ST_ differentiation values and statistical significance was determined by running 1000 permutations of the haplotype matrix. The minimum geographic distance between pairs of locations was measured in kilometres using the path ruler tool in Google Earth, from a height of 20 km.

Populations (Supplementary Table S6) were divided in seven groups according to the dispersal simulation (see dispersal potential simulation below). These were: Eastern Mediterranean (EM; populations KR, BZ), Western Mediterranean (WM; population AN), Alboran Sea (AS; populations PN, CG, BM, AM, LA, TG and LP), Atlantic Iberia (AI; populations AT, PM, TV, VL and SG), Northwestern Morocco (NM; populations LR, RB, CB, SB, EB and ES), Southern Morocco (SM; IM, ML, TT and BJ) and Western Sahara (WS; population LB and DK).

To evaluate population genetic structure, a hierarchical analysis of molecular variance (AMOVA) was conducted in Arlequin 3.11 using 10,100 permutations. To understand how genetic variation is partitioned between distinct groups, among locations within groups and within locations, the seven groups described above were designated *a priori* (EM, WM, AS, IB, NM, SM and WS).

Intra-specific genealogical relationships between groups and the relative frequency of haplotypes were determined by a median-joining haplotype network built on Network 4.5.0.2^99^.

Microsatellite markers: Allele sizes were scored using STRAND software (http://www.vgl.ucdavis.edu/informatics/STRand), binned with the StandArich package in R 2.10.1 software and manually reviewed for ambiguities. MICRO-CHECKER^100^ was used to test for stuttering, null alleles and large allele dropout at each locus and population. All loci had less than 5% of missing data with the exception of P16 (22%), which was excluded from the following analyses.

Observed (H_O_) and expected (H_E_) heterozygosity, inbreeding coefficient (F_IS_^101^) and allelic richness (Â) were estimated for each population. Allelic richness (Â) was additionally standardized to two of the smallest sample sizes using GENETIX 4.05^102^ and FSTAT^103^. Allelic frequencies for each marker and population were plotted using StandArich. Deviations from Hardy-Weinberg equilibrium were tested running 10,000 permutations using GENETIX. Pairwise genetic differentiation was estimated as F_ST_^101^ and as Jost’s D^104^ using diveRsity^105^ in R, with significance tested 1,000 bootstrap. Pairwise genetic distance between populations was estimated based on the proportion of shared alleles^106^ according to^107^ using Populations 1.2.30^108^ testing significance with 999 bootstrap. A neighbour-joining (NJ) phylogenetic tree was constructed using MEGA5^109^.

STRUCTURE 2.3.4 software^110^ estimated population structure and inferred the number of clusters in the dataset, considering no prior information on populations. An admixture model and correlated allele frequencies were assumed. The number of possible clusters (K) assessed ranged from 1 to 28 (maximum number of populations plus one) and five independent runs with 100,000 Markov Chain Monte Carlo (MCMC) iterations and 50,000 burn-in were performed for each K. STRUCTURE HARVESTER^111^ and CLUMPP^112^ estimated the most probable number of Ks (clusters) and found the consensus of the five replicated runs for the selected K respectively. The replicate consensus was plotted with Ruby package Bar Plotter (http://evolution.unibas.ch/salzburger/software.htm). A discriminant analysis of principal components (DAPC) was performed in R with *adegenet* ^113^ based on the matrix of individual genotypes with seven microsatellite loci, to characterize the genetic variation of the study area. Each individual was assigned to its sampling population. The function *find*.*cluster* returned the best fitting number of clusters (K) in the dataset, correspondent to the minimum K after which the Bayesian Information Criterion changes by a negligible amount^114^.

A hierarchical analysis of molecular variance (AMOVA) was conducted in Arlequin 3.11 running 10,100 permutations, to understand hierarchical population structure with groups designated *a priori* based on potential oceanographic barriers as described above.

#### Dispersal potential simulation

Lagrangian particle simulations (LPS; as in ref. 6) were performed in R using the packages data.table, dismo, parallel, raster and vegan to infer the dispersal potential of *P*. *perna* throughout its northeastern Atlantic and Mediterranean range distribution. The simulations used high-resolution data of ocean currents assembled from the Hybrid Coordinate Ocean Model (HYCOM). The coastlines of the northeastern Atlantic and Mediterranean region (comprising ~6500 km) were gridded to cells with a common spatial resolution of 0.01° (9828 cells in total), from which individual passive particles simulating pelagic states of *P*. *perna* were released every 12 hours, from February to June (spawning period of *P*. *perna* ^85, 115^). The particles were allowed to drift in the simulated environment for a maximum period of 30 days^6^ until ending up on shore. The position of each individual particle was computed every hour using the bilinear interpolation of HYCOM’s daily velocity fields. The final particle trajectories were used to estimate the asymmetrical connectivity between all pairs of coastline cells, by averaging the time taken (days) of all particles released from cell i that ended up on cell j. Inter-annual variability in ocean currents was taken into account by averaging simulations running independently per year, for a period of 11 years (2002 to 2012).

A network analysis (also referred to as graph-theoretical analysis) was performed in R using the igraph package to consider a year to year stepping-stone scenario^18^. The Floyd-Warshall’s algorithm found the shortest path between every pair of cells, by using the transport time from the averaged connectivity matrix as the relative asymmetrical weight. The transport times found throughout the shortest paths were summed to produce a stepping-stone connectivity matrix between all pairs of cells. This matrix allowed extracting connectivity estimates between the sites sampled for genetics. A Linear regression model tested the pairwise genetic distance as FST/(1 − FST) against the minimum transport time between sites, on the stepping-stone scenario. A null model was performed with genetic distances against shortest marine distances. Adjusted R-squared and Akaike Information Criteria (AIC) were adopted to infer the efficiency of each model in explaining genetic differentiation.

The stepping-stone connectivity matrix was further used to identify major oceanographic regions. To this end, network percolation was performed to remove the weak connections between cells until a threshold was reached that allowed all cells to be connected into a unique network^116^, that maximized modularity, an index that quantifies the goodness of fit of a given network^117^. This approach removed surplus connections with irrelevant information. The leading eigenvector algorithm^117^, commonly used to detect communities in networks^118^, was then used to assign a unique membership to the nodes (i.e. coastal cells) of the percolated network. In practice this allowed the delineation of unique oceanographic regions structured by ocean currents, where the likelihood of cells being connected within the same region (i.e. membership) is higher than the likelihood of connection among regions. The statistical significance of this step was inferred by testing the proportion of 1e10^4^ membership assignments performed randomly that retrieved a modularity higher than observed. Finally, the strength of barriers separating each oceanographic region was quantified by dividing the absolute number of coastal cells connecting the regions *i* with *j* on at least one dispersal event, by the absolute number of connected cells within region *i*, and multiplying this by 100 so that the strength of barriers would be reflected as % reduction in connectivity.

### Ecological niche modelling of *P*. *perna* native distribution

#### Data on native occurrence

A total of 118 presences were compiled from extensive field surveys and from records in the existing literature where the species is native (i.e. the African continent, southern Iberia and the Arabian Peninsula). As the number of true absences detected in the field was relatively low (52) and biased towards the areas where most of the field surveys are available (i.e. South Africa, Morocco and the Iberian Peninsula), pseudo-absences from non-surveyed areas were randomly added and included in the models (Supplementary Table S7). The use of pseudo-absences is particularly useful as a surrogate for accurate absence data to perform presence-absence models; pseudo-absence models also avoid overoptimistic predictions, a common characteristic of presence-only approaches^119, 120^. The selection method to generate pseudo-absence data conditions the predictions of the model^119, 120^. By performing a random selection of pseudo-absences the modelled range is not overpredicted, allowing a better assessment of the variables affecting the realized distribution of the species^119^.

The intertidal area was delimited by extracting the coastal cells covering a range from −2 to 1 m from the General Bathymetric Chart of the Oceans (GEBCO) gridded bathymetric data set with a spatial resolution of 30 arc-seconds (http://www.gebco.net/).

#### Environmental variables

The most meaningful environmental variables commonly known to influence and used to model the distribution of intertidal species^4, 6, 36^ were obtained from Bio-ORACLE dataset^121^ at a spatial resolution of 5 arcmin (9.2 km). These included minimum and maximum surface air temperature (SAT)^122^, minimum and maximum sea surface temperature (SST), nutrients (nitrate and phosphate concentrations), salinity and mean cloud cover fraction (Supplementary Table S8). Significant wave height (2009–2015) was obtained from Aviso (http://www.aviso.altimetry.fr; Supplementary Table S8). All variables and species’ records were georeferenced to the same resolution (9.2 km). Correlation among predictors was verified using Pearson’s correlation coefficient = |0.7| as a cut-off.

#### Niche modelling and variable importance

The environmental niche of *P*. *perna* was modelled using six presence-absence techniques: generalized additive model (GAM), generalized boosting model (GBM), generalized linear model (GLM), flexible discriminant analysis (FDA), randomForest (RF), and multiple adaptive regression splines (MARS) using biomod2 package^123^ in R. Pseudo-absences were selected at random to complement absence data surveyed in the field. A proportion presence/absence of 1:10 (as in ref. 35) corresponding to a total amount of 1180 absences (52 true absences +1128 pseudo-absences) was used, for a study region of 20,057 cells of coastal areas. Cross-validation was performed by randomly splitting the data records into training (70%) and test (30%) datasets. Moreover, the area under the receiver operating characteristic (ROC) curve (AUC), the ROC-derived sensitivity and specificity values^124^, and the true skill statistic (TSS^125^), using the threshold which optimized ROC and TSS scores^123^, were used to evaluate the models. Each of the six algorithms ran 50 iterations and a “committee averaging” ensemble model was performed averaging the binary predictions of models with TSS > 0.7 to predict the probability of occurrence of the species.

The relative contribution of each variable was calculated by estimating the correlation between each model without a variable and the full model^126^, running three permutations. The subtraction of 1 minus the correlation was calculated and each predictor was scored 0–1 (lowest to highest importance)^123^. Subsequently, a mean of the scores of the three permutations was calculated for each variable.

### Data analysis

To disentangle the individual significance of minimum SST and SAT on *P*. *perna* distribution, two subsets of nine locations equitably distributed along approximately 2,200 km of the cool temperate southwest (CT) and the warm temperate (WT) southern African provinces^31^ were selected. *Perna perna* has been extensively investigated along these shores where a wide distributional gap has been described (*P*. *perna* is absent from CT but present in WT)^32^. The contrasting distributional pattern of *P*. *perna* along the CT and the WT regions coupled to SST and SAT data allows a better understanding of the environmental factors setting the distributional limits of this species. While all nine absence records from the CT were included, three different subsets of nine locations along WT were selected. The extents of coastlines and the distances between locations were estimated on Google Earth at an altitude of 100 m.

One-way ANOVA was used to test the null hypothesis that minimum SST and minimum SAT did not differ significantly between the two regions in South Africa where *P*. *perna* is absent or present. The design consisted of one factor: Record (two levels, fixed) and the analyses were performed three times. All tests and respective significance values were performed with STATISTICA (StatSoft). When data did not fulfil the pre-requisites for parametric analysis (Cochran’s Test or Shapiro-Wilk’s W), analyses were performed using PERMANOVA^127, 128^ running 999 permutations.

### Data availability

The mitochondrial and nuclear genetic data generated and analysed during the current study have been deposited in GenBank (accession numbers KY514494 - KY515223) and can be found in Supplementary Table S9, respectively.

## Electronic supplementary material

Supplementary Information
