# Supplementary material for: Evidence for rangewide panmixia despite multiple barriers to dispersal in a marine mussel
Source: Sci Rep. 2017 Aug 31;7:10279. doi: 10.1038/s41598-017-10753-9 (PMC5579014; doi:10.1038/s41598-017-10753-9)
Supplement: Supplementary file 1 — Supplementary Information [file 41598_2017_10753_MOESM1_ESM.pdf]

Evidence for rangewide panmixia despite barriers to dispersal in a marine mussel

Scientific Reports

Supplementary information

Carla R. Lourenço*[1,2], Katy R. Nicastro[1], Christopher D. McQuaid[2], Rosa M. Chefaoui[1], Jorge Assis[1], Mohammed Z. Taleb[3], Gerardo I. Zardi[2]

[1]CCMAR-CIMAR – Associated Laboratory, University of Algarve, Campus de Gambelas, Faro 8005-139, Portugal

[2]Department of Zoology and Entomology, Rhodes University, Grahamstown 6140, South Africa

[3]Department of Biology, Faculty of Natural and Life Sciences, University of Oran Ahmed Ben Bella, 31000 Oran, Algeria

*Correspondence: Carla R. Lourenço, CCMAR-CIMAR – Associated Laboratory, University of Algarve, Campus de Gambelas, Faro 8005-139, Portugal.

E-mail: carla.rodrigues.lourenco@gmail.com

Table S1 - Pairwise ϕ_{ST} comparison of *Perna perna* populations. All P-values were corrected and non-significant. Locations codes as in Table S6.

	KR	BZ	AN	PN	CG	BM	AM	LA	TG	LP	AT	PM	TV	VL	SG	LR	RB	CB	SB	EB	ES	IM	ML	TT	BJ	LB	DK
KR	-	0.000	0.008	0.000	0.000	0.000	0.025	0.000	0.013	0.195	0.017	0.027	0.000	0.000	0.127	0.000	0.000	0.000	0.000	0.011	0.013	0.030	0.000	0.014	0.029	0.000	0.000
BZ		-	0.000	0.000	0.000	0.000	0.006	0.000	0.000	0.115	0.000	0.002	0.000	0.000	0.121	0.000	0.000	0.000	0.000	0.000	0.008	0.000	0.000	0.000	0.000	0.000	0.000
AN			-	0.000	0.000	0.000	0.026	0.006	0.000	0.216	0.014	0.058	0.029	0.015	0.256	0.020	0.000	0.002	0.000	0.011	0.054	0.019	0.026	0.020	0.026	0.000	0.000
PN				-	0.000	0.000	0.000	0.000	0.000	0.114	0.000	0.000	0.000	0.000	0.155	0.002	0.000	0.000	0.000	0.000	0.004	0.000	0.000	0.000	0.000	0.000	0.002
CG					-	0.000	0.006	0.000	0.000	0.082	0.008	0.085	0.000	0.014	0.236	0.017	0.000	0.000	0.000	0.024	0.037	0.043	0.003	0.026	0.040	0.000	0.000
BM						-	0.020	0.001	0.000	0.219	0.004	0.025	0.018	0.002	0.219	0.007	0.000	0.000	0.000	0.002	0.033	0.005	0.014	0.006	0.010	0.000	0.000
AM							-	0.000	0.009	0.153	0.000	0.012	0.000	0.013	0.255	0.018	0.000	0.000	0.000	0.002	0.000	0.015	0.000	0.000	0.000	0.022	0.037
LA								-	0.000	0.039	0.000	0.014	0.000	0.000	0.127	0.000	0.000	0.000	0.000	0.001	0.000	0.017	0.000	0.000	0.004	0.000	0.000
TG									-	0.142	0.000	0.046	0.013	0.020	0.234	0.025	0.000	0.000	0.000	0.012	0.032	0.011	0.015	0.002	0.016	0.000	0.012
LP										-	0.252	0.432	0.063	0.212	0.201	0.087	0.156	0.197	0.092	0.225	0.167	0.390	0.128	0.225	0.350	0.229	0.158
AT											-	0.005	0.000	0.000	0.300	0.008	0.000	0.000	0.000	0.000	0.000	0.000	0.000	0.000	0.000	0.005	0.026
PM												-	0.014	0.000	0.324	0.000	0.005	0.003	0.008	0.000	0.000	0.005	0.006	0.000	0.000	0.026	0.044
TV													-	0.000	0.113	0.000	0.000	0.000	0.000	0.006	0.000	0.031	0.000	0.003	0.015	0.010	0.008
VL														-	0.138	0.000	0.000	0.000	0.000	0.000	0.000	0.006	0.000	0.000	0.006	0.000	0.000
SG															-	0.048	0.192	0.189	0.146	0.210	0.172	0.349	0.177	0.217	0.332	0.196	0.113
LR																-	0.000	0.000	0.000	0.001	0.000	0.013	0.000	0.004	0.012	0.000	0.000
RB																	-	0.000	0.000	0.000	0.006	0.000	0.000	0.000	0.000	0.000	0.003
CB																		-	0.000	0.000	0.000	0.000	0.000	0.000	0.000	0.000	0.000
SB																			-	0.000	0.001	0.006	0.000	0.000	0.000	0.000	0.002
EB																				-	0.005	0.000	0.000	0.000	0.000	0.002	0.020
ES																					-	0.028	0.000	0.000	0.005	0.028	0.031
IM																						-	0.020	0.000	0.000	0.009	0.038
ML																							-	0.001	0.005	0.011	0.013
TT																								-	0.000	0.006	0.027
BJ																									-	0.015	0.041
LB																										-	0.000
DK																											-

Table S2 – a) Pairwise F_{ST} (above) and Jost's Dest (below) comparisons of *P. perna* populations. Locations codes as in Table S6.

a)	KR	BZ	OR	PN	CG	BM	AM	LA	TG	LP	AT	PM	TV	VL	SG	LR	RB	CB	SB	EB	ES	IM	ML	TT	BJ	LB	DK
KR		0.000	0.001	0.001	0.000	0.000	0.001	0.000	0.000	0.000	0.000	0.000	0.001	0.000	0.021	0.000	0.000	0.000	0.002	0.000	0.000	0.004	0.004	0.000	0.001	0.000	0.000
BZ	0.000		0.000	0.003	0.000	0.000	0.000	0.000	0.002	0.000	0.000	0.000	0.000	0.000	0.019	0.000	0.000	0.001	0.000	0.000	0.000	0.000	0.000	0.000	0.000	0.000	0.000
OR	0.001	0.004		0.000	0.000	0.000	0.000	0.000	0.000	0.000	0.000	0.000	0.009	0.000	0.006	0.001	0.000	0.000	0.000	0.000	0.000	0.003	0.006	0.003	0.002	0.000	0.000
PN	0.007	0.016	0.002		0.000	0.001	0.000	0.000	0.001	0.000	0.000	0.006	0.011	0.001	0.012	0.002	0.003	0.000	0.009	0.004	0.001	0.010	0.011	0.003	0.006	0.003	0.000
CG	0.005	0.000	0.000	0.003		0.000	0.000	0.000	0.000	0.000	0.000	0.000	0.000	0.000	0.012	0.000	0.000	0.000	0.000	0.000	0.000	0.000	0.000	0.000	0.000	0.000	0.000
BM	0.000	0.000	0.000	0.005	0.001		0.000	0.000	0.000	0.000	0.000	0.000	0.004	0.000	0.005	0.000	0.000	0.000	0.000	0.000	0.000	0.000	0.001	0.000	0.007	0.000	0.000
AM	0.000	0.000	0.002	0.001	0.001	0.000		0.000	0.002	0.000	0.000	0.000	0.000	0.000	0.005	0.000	0.000	0.000	0.001	0.002	0.000	0.001	0.000	0.000	0.005	0.000	0.000
LA	0.000	0.000	0.000	0.000	0.001	0.000	0.000		0.000	0.000	0.000	0.000	0.004	0.000	0.005	0.000	0.000	0.000	0.000	0.000	0.000	0.001	0.000	0.000	0.002	0.000	0.000
TG	0.000	0.000	0.000	0.004	0.003	0.000	0.000	0.000		0.000	0.003	0.003	0.005	0.000	0.021	0.000	0.004	0.001	0.001	0.000	0.000	0.007	0.004	0.001	0.001	0.000	0.002
LP	0.000	0.000	0.000	0.000	0.000	0.000	0.000	0.000	0.000		0.000	0.000	0.000	0.000	0.000	0.000	0.000	0.000	0.000	0.000	0.000	0.000	0.000	0.000	0.000	0.000	0.000
AT	0.000	0.000	0.000	0.000	0.000	0.000	0.000	0.000	0.000	0.000		0.000	0.002	0.000	0.008	0.000	0.000	0.000	0.003	0.000	0.002	0.000	0.002	0.000	0.007	0.000	0.000
PM	0.000	0.000	0.001	0.002	0.002	0.000	0.000	0.001	0.003	0.000	0.000		0.000	0.000	0.024	0.000	0.000	0.005	0.000	0.000	0.000	0.000	0.000	0.000	0.000	0.000	0.000
TV	0.001	0.000	0.013	0.039	0.003	0.003	0.001	0.006	0.019	0.000	0.000	0.007		0.000	0.027	0.000	0.002	0.003	0.006	0.009	0.002	0.003	0.000	0.000	0.001	0.000	0.007
VL	0.000	0.000	0.004	0.001	0.007	0.000	0.000	0.000	0.000	0.000	0.000	0.000	0.000		0.024	0.000	0.000	0.000	0.001	0.000	0.000	0.004	0.000	0.000	0.000	0.000	0.001
SG	0.001	0.000	0.001	0.008	0.001	0.000	0.000	0.000	0.000	0.000	0.000	0.006	0.000	0.008		0.009	0.009	0.000	0.003	0.010	0.018	0.025	0.024	0.018	0.034	0.021	0.011
LR	0.000	0.001	0.000	0.004	0.000	0.000	0.000	0.000	0.001	0.000	0.000	0.000	0.002	0.000	0.000		0.000	0.000	0.000	0.000	0.001	0.001	0.000	0.000	0.002	0.000	0.000
RB	0.001	0.000	0.001	0.014	0.000	0.000	0.000	0.000	0.005	0.000	0.000	0.005	0.000	0.007	0.000	0.001		0.000	0.001	0.000	0.000	0.000	0.000	0.000	0.003	0.000	0.000
CB	0.000	0.003	0.001	0.009	0.002	0.000	0.000	0.000	0.000	0.000	0.000	0.001	0.003	0.000	0.000	0.000	0.000		0.005	0.001	0.003	0.007	0.005	0.000	0.007	0.000	0.000
SB	0.003	0.001	0.004	0.010	0.000	0.002	0.011	0.000	0.000	0.000	0.000	0.002	0.028	0.000	0.000	0.000	0.008	0.015		0.000	0.000	0.001	0.000	0.000	0.000	0.000	0.003
EB	0.002	0.004	0.000	0.017	0.006	0.001	0.006	0.000	0.000	0.000	0.000	0.003	0.041	0.003	0.003	0.002	0.000	0.006	0.000		0.002	0.001	0.001	0.000	0.005	0.000	0.000
ES	0.001	0.000	0.000	0.000	0.000	0.000	0.000	0.000	0.000	0.000	0.000	0.000	0.007	0.000	0.000	0.000	0.000	0.001	0.000	0.002		0.003	0.001	0.001	0.000	0.000	0.000
IM	0.002	0.001	0.003	0.026	0.002	0.000	0.007	0.001	0.008	0.000	0.000	0.000	0.002	0.013	0.002	0.002	0.000	0.009	0.006	0.005	0.012		0.000	0.000	0.007	0.000	0.003
ML	0.004	0.000	0.015	0.007	0.002	0.000	0.000	0.000	0.000	0.000	0.000	0.000	0.000	0.000	0.009	0.000	0.003	0.006	0.000	0.004	0.000	0.003		0.000	0.004	0.000	0.002
TT	0.000	0.000	0.007	0.000	0.001	0.000	0.000	0.000	0.000	0.000	0.000	0.000	0.004	0.004	0.000	0.000	0.000	0.001	0.000	0.007	0.006	0.000	0.000		0.003	0.000	0.000
BJ	0.005	0.002	0.000	0.014	0.002	0.012	0.012	0.004	0.006	0.000	0.003	0.004	0.009	0.004	0.008	0.002	0.006	0.010	0.001	0.013	0.001	0.016	0.016	0.010		0.000	0.006
LB	0.000	0.001	0.000	0.014	0.000	0.000	0.000	0.000	0.000	0.000	0.000	0.002	0.002	0.000	0.005	0.000	0.000	0.000	0.003	0.000	0.000	0.001	0.003	0.000	0.000		0.000
DK	0.002	0.001	0.002	0.004	0.001	0.000	0.000	0.000	0.000	0.000	0.000	0.000	0.027	0.002	0.002	0.005	0.001	0.001	0.000	0.000	0.000	0.010	0.002	0.000	0.015	0.001	

Table S2 – b) 95% Confidence intervals of pairwise F_{ST} (above) and Jost's Dest (below) comparisons of *P. perna* populations. Locations codes as in Table S6.

	KR	BZ	OR	PN	CG	BM	AM	LA	TG	LP	AT	PM	TV	VL	SG	LR	RB	CB	SB	EB	ES	IM	ML	TT	BJ	LB	DK
KR		(-0.014-0.012)	(-0.013-0.022)	(-0.011-0.017)	(-0.021-0.021)	(-0.010-0.014)	(-0.010-0.015)	(-0.014-0.010)	(-0.013-0.009)	(-0.097-0.076)	(-0.013-0.016)	(-0.016-0.019)	(-0.011-0.015)	(-0.014-0.011)	(-0.031-0.100)	(-0.015-0.009)	(-0.011-0.015)	(-0.013-0.014)	(-0.011-0.018)	(-0.013-0.011)	(-0.012-0.013)	(-0.008-0.021)	(-0.008-0.023)	(-0.014-0.010)	(-0.010-0.017)	(-0.013-0.008)	(-0.012-0.016)
BZ	(-0.027-0.046)		(-0.015-0.016)	(-0.010-0.021)	(-0.025-0.017)	(-0.011-0.012)	(-0.012-0.012)	(-0.016-0.010)	(-0.009-0.015)	(-0.101-0.078)	(-0.012-0.017)	(-0.017-0.010)	(-0.014-0.013)	(-0.013-0.010)	(-0.037-0.109)	(-0.013-0.013)	(-0.014-0.010)	(-0.013-0.020)	(-0.013-0.011)	(-0.012-0.013)	(-0.013-0.010)	(-0.012-0.013)	(-0.014-0.008)	(-0.014-0.008)	(-0.013-0.010)	(-0.015-0.010)	(-0.013-0.015)
OR	(-0.035-0.062)	(-0.023-0.049)		(-0.016-0.018)	(-0.029-0.020)	(-0.016-0.012)	(-0.013-0.018)	(-0.019-0.009)	(-0.015-0.020)	(-0.089-0.105)	(-0.016-0.016)	(-0.016-0.020)	(-0.009-0.033)	(-0.014-0.020)	(-0.050-0.102)	(-0.014-0.021)	(-0.016-0.011)	(-0.016-0.015)	(-0.012-0.015)	(-0.016-0.014)	(-0.014-0.019)	(-0.009-0.021)	(-0.008-0.025)	(-0.010-0.023)	(-0.013-0.025)	(-0.018-0.016)	(-0.016-0.017)
PN	(-0.028-0.062)	(-0.024-0.071)	(-0.028-0.052)		(-0.021-0.030)	(-0.010-0.016)	(-0.012-0.013)	(-0.013-0.011)	(-0.010-0.016)	(-0.096-0.080)	(-0.012-0.015)	(-0.011-0.029)	(-0.004-0.031)	(-0.012-0.020)	(-0.045-0.109)	(-0.010-0.020)	(-0.008-0.016)	(-0.011-0.015)	(-0.005-0.026)	(-0.008-0.021)	(-0.010-0.018)	(-0.004-0.029)	(-0.004-0.031)	(-0.009-0.019)	(-0.008-0.026)	(-0.009-0.021)	(-0.012-0.015)
CG	(-0.039-0.080)	(-0.039-0.074)	(-0.043-0.070)	(-0.044-0.088)		(-0.022-0.023)	(-0.026-0.020)	(-0.023-0.020)	(-0.018-0.025)	(-0.121-0.101)	(-0.026-0.015)	(-0.026-0.021)	(-0.023-0.023)	(-0.020-0.021)	(-0.051-0.108)	(-0.024-0.018)	(-0.025-0.014)	(-0.022-0.022)	(-0.025-0.018)	(-0.017-0.022)	(-0.023-0.019)	(-0.020-0.024)	(-0.021-0.024)	(-0.022-0.019)	(-0.021-0.022)	(-0.023-0.019)	(-0.026-0.024)
BM	(-0.034-0.044)	(-0.024-0.043)	(-0.026-0.045)	(-0.026-0.053)	(-0.047-0.078)		(-0.013-0.009)	(-0.014-0.006)	(-0.011-0.016)	(-0.083-0.091)	(-0.015-0.008)	(-0.015-0.018)	(-0.008-0.020)	(-0.011-0.013)	(-0.048-0.093)	(-0.013-0.010)	(-0.012-0.010)	(-0.013-0.014)	(-0.011-0.011)	(-0.011-0.011)	(-0.011-0.012)	(-0.013-0.010)	(-0.010-0.015)	(-0.014-0.011)	(-0.007-0.024)	(-0.015-0.010)	(-0.013-0.011)
AM	(-0.036-0.052)	(-0.031-0.043)	(-0.031-0.055)	(-0.030-0.043)	(-0.042-0.077)	(-0.037-0.036)		(-0.014-0.007)	(-0.009-0.017)	(-0.106-0.077)	(-0.016-0.010)	(-0.014-0.020)	(-0.013-0.016)	(-0.016-0.009)	(-0.051-0.098)	(-0.013-0.011)	(-0.012-0.009)	(-0.014-0.012)	(-0.010-0.016)	(-0.009-0.017)	(-0.015-0.009)	(-0.011-0.016)	(-0.012-0.013)	(-0.014-0.009)	(-0.008-0.022)	(-0.014-0.009)	(-0.015-0.011)
LA	(-0.022-0.040)	(-0.027-0.038)	(-0.023-0.039)	(-0.030-0.044)	(-0.038-0.071)	(-0.038-0.022)	(-0.040-0.028)		(-0.013-0.014)	(-0.095-0.080)	(-0.013-0.013)	(-0.014-0.020)	(-0.010-0.022)	(-0.015-0.012)	(-0.046-0.092)	(-0.014-0.010)	(-0.014-0.007)	(-0.015-0.008)	(-0.014-0.008)	(-0.014-0.010)	(-0.015-0.011)	(-0.010-0.015)	(-0.013-0.015)	(-0.013-0.011)	(-0.011-0.020)	(-0.015-0.012)	(-0.015-0.010)
TG	(-0.031-0.045)	(-0.024-0.056)	(-0.030-0.048)	(-0.028-0.051)	(-0.044-0.082)	(-0.037-0.045)	(-0.037-0.050)	(-0.040-0.035)		(-0.108-0.065)	(-0.010-0.020)	(-0.011-0.023)	(-0.008-0.021)	(-0.013-0.012)	(-0.033-0.117)	(-0.011-0.013)	(-0.007-0.019)	(-0.011-0.017)	(-0.012-0.020)	(-0.013-0.010)	(-0.011-0.015)	(-0.007-0.025)	(-0.009-0.021)	(-0.009-0.013)	(-0.011-0.018)	(-0.013-0.010)	(-0.011-0.018)
LP	(-0.085-0.194)	(-0.093-0.194)	(-0.094-0.248)	(-0.075-0.175)	(-0.102-0.230)	(-0.101-0.227)	(-0.088-0.205)	(-0.100-0.203)	(-0.084-0.149)		(-0.082-0.082)	(-0.105-0.095)	(-0.082-0.088)	(-0.084-0.086)	(-0.174-0.209)	(-0.087-0.080)	(-0.089-0.085)	(-0.097-0.064)	(-0.089-0.082)	(-0.096-0.079)	(-0.091-0.092)	(-0.080-0.112)	(-0.080-0.093)	(-0.083-0.090)	(-0.091-0.077)	(-0.08-0.093)	(-0.105-0.090)
AT	(-0.041-0.058)	(-0.036-0.054)	(-0.039-0.062)	(-0.039-0.048)	(-0.047-0.066)	(-0.050-0.029)	(-0.052-0.031)	(-0.042-0.044)	(-0.044-0.058)	(-0.101-0.214)		(-0.015-0.022)	(-0.012-0.020)	(-0.013-0.013)	(-0.044-0.094)	(-0.017-0.006)	(-0.014-0.009)	(-0.016-0.011)	(-0.010-0.018)	(-0.012-0.014)	(-0.012-0.018)	(-0.013-0.017)	(-0.010-0.021)	(-0.014-0.015)	(-0.007-0.027)	(-0.016-0.011)	(-0.014-0.015)
PM	(-0.033-0.053)	(-0.042-0.033)	(-0.031-0.055)	(-0.042-0.063)	(-0.045-0.086)	(-0.036-0.061)	(-0.044-0.058)	(-0.035-0.056)	(-0.038-0.062)	(-0.101-0.243)	(-0.041-0.064)		(-0.015-0.019)	(-0.016-0.019)	(-0.033-0.106)	(-0.015-0.021)	(-0.014-0.018)	(-0.013-0.031)	(-0.013-0.017)	(-0.014-0.017)	(-0.016-0.015)	(-0.016-0.015)	(-0.014-0.014)	(-0.015-0.016)	(-0.014-0.015)	(-0.017-0.017)	(-0.017-0.021)
TV	(-0.038-0.056)	(-0.030-0.046)	(-0.037-0.088)	(-0.007-0.096)	(-0.044-0.080)	(-0.037-0.062)	(-0.032-0.052)	(-0.028-0.064)	(-0.021-0.072)	(-0.089-0.212)	(-0.043-0.066)	(-0.034-0.065)		(-0.015-0.012)	(-0.026-0.112)	(-0.013-0.013)	(-0.011-0.018)	(-0.011-0.024)	(-0.007-0.022)	(-0.005-0.027)	(-0.010-0.016)	(-0.011-0.021)	(-0.014-0.012)	(-0.013-0.015)	(-0.011-0.017)	(-0.014-0.014)	(-0.009-0.027)
VL	(-0.032-0.049)	(-0.030-0.044)	(-0.037-0.067)	(-0.040-0.053)	(-0.041-0.078)	(-0.031-0.046)	(-0.034-0.039)	(-0.038-0.047)	(-0.038-0.050)	(-0.095-0.209)	(-0.049-0.049)	(-0.043-0.060)	(-0.034-0.052)		(-0.029-0.120)	(-0.015-0.011)	(-0.010-0.013)	(-0.016-0.012)	(-0.010-0.015)	(-0.012-0.012)	(-0.016-0.008)	(-0.008-0.019)	(-0.014-0.015)	(-0.011-0.013)	(-0.012-0.015)	(-0.017-0.005)	(-0.012-0.017)

SG	(-0.167-0.220)	(-0.157-0.221)	(-0.121-0.225)	(-0.118-0.263)	(-0.141-0.226)	(-0.136-0.193)	(-0.133-0.220)	(-0.138-0.213)	(-0.158-0.241)	(-0.175-0.279)	(-0.135-0.228)	(-0.153-0.222)	(-0.179-0.213)	(-0.152-0.236)		(-0.041-0.090)	(-0.042-0.092)	(-0.052-0.087)	(-0.050-0.084)	(-0.043-0.093)	(-0.037-0.109)	(-0.032-0.110)	(-0.028-0.110)	(-0.037-0.104)	(-0.021-0.119)	(-0.034-0.112)	(-0.046-0.108)
LR	(-0.027-0.053)	(-0.029-0.052)	(-0.043-0.070)	(-0.038-0.072)	(-0.046-0.080)	(-0.032-0.054)	(-0.036-0.048)	(-0.028-0.049)	(-0.032-0.048)	(-0.106-0.228)	(-0.058-0.025)	(-0.044-0.066)	(-0.032-0.053)	(-0.044-0.051)	(-0.144-0.213)		(-0.013-0.011)	(-0.014-0.011)	(-0.012-0.013)	(-0.012-0.010)	(-0.010-0.016)	(-0.011-0.018)	(-0.014-0.012)	(-0.015-0.007)	(-0.011-0.018)	(-0.016-0.007)	(-0.013-0.018)
RB	(-0.027-0.048)	(-0.021-0.037)	(-0.025-0.043)	(-0.017-0.058)	(-0.035-0.064)	(-0.023-0.036)	(-0.023-0.036)	(-0.019-0.032)	(-0.026-0.047)	(-0.088-0.224)	(-0.027-0.037)	(-0.030-0.059)	(-0.038-0.056)	(-0.024-0.052)	(-0.142-0.221)	(-0.026-0.050)		(-0.016-0.010)	(-0.008-0.015)	(-0.011-0.010)	(-0.010-0.014)	(-0.012-0.014)	(-0.011-0.013)	(-0.013-0.012)	(-0.010-0.020)	(-0.014-0.010)	(-0.014-0.010)
CB	(-0.036-0.060)	(-0.035-0.060)	(-0.036-0.057)	(-0.025-0.058)	(-0.050-0.077)	(-0.041-0.050)	(-0.036-0.042)	(-0.026-0.047)	(-0.038-0.054)	(-0.093-0.188)	(-0.049-0.044)	(-0.049-0.074)	(-0.041-0.064)	(-0.053-0.041)	(-0.135-0.234)	(-0.032-0.059)	(-0.027-0.046)		(-0.007-0.022)	(-0.010-0.015)	(-0.011-0.020)	(-0.007-0.027)	(-0.008-0.023)	(-0.012-0.016)	(-0.008-0.027)	(-0.018-0.009)	(-0.014-0.015)
SB	(-0.034-0.056)	(-0.026-0.040)	(-0.027-0.051)	(-0.041-0.073)	(-0.044-0.064)	(-0.032-0.047)	(-0.028-0.067)	(-0.035-0.035)	(-0.038-0.060)	(-0.098-0.225)	(-0.048-0.060)	(-0.037-0.056)	(-0.017-0.081)	(-0.038-0.053)	(-0.123-0.217)	(-0.040-0.053)	(-0.020-0.050)	(-0.025-0.082)		(-0.013-0.009)	(-0.012-0.013)	(-0.009-0.014)	(-0.011-0.011)	(-0.011-0.014)	(-0.012-0.019)	(-0.012-0.017)	(-0.009-0.021)
EB	(-0.024-0.045)	(-0.025-0.049)	(-0.028-0.052)	(-0.016-0.064)	(-0.040-0.077)	(-0.026-0.039)	(-0.029-0.054)	(-0.027-0.040)	(-0.023-0.035)	(-0.080-0.202)	(-0.035-0.053)	(-0.032-0.057)	(-0.007-0.105)	(-0.030-0.045)	(-0.124-0.202)	(-0.027-0.047)	(-0.026-0.041)	(-0.029-0.058)	(-0.031-0.042)		(-0.010-0.017)	(-0.009-0.014)	(-0.009-0.014)	(-0.009-0.015)	(-0.007-0.023)	(-0.013-0.010)	(-0.012-0.016)
ES	(-0.030-0.044)	(-0.025-0.036)	(-0.039-0.056)	(-0.035-0.045)	(-0.048-0.071)	(-0.035-0.045)	(-0.047-0.029)	(-0.048-0.026)	(-0.036-0.050)	(-0.089-0.218)	(-0.053-0.056)	(-0.039-0.048)	(-0.029-0.049)	(-0.045-0.029)	(-0.147-0.222)	(-0.043-0.065)	(-0.030-0.043)	(-0.043-0.060)	(-0.034-0.048)	(-0.032-0.050)		(-0.009-0.020)	(-0.011-0.016)	(-0.010-0.016)	(-0.011-0.016)	(-0.014-0.010)	(-0.012-0.018)
IM	(-0.030-0.053)	(-0.024-0.041)	(-0.029-0.062)	(-0.016-0.077)	(-0.042-0.076)	(-0.029-0.040)	(-0.029-0.053)	(-0.027-0.043)	(-0.031-0.063)	(-0.100-0.241)	(-0.036-0.049)	(-0.030-0.057)	(-0.038-0.056)	(-0.019-0.056)	(-0.149-0.196)	(-0.029-0.057)	(-0.022-0.035)	(-0.032-0.063)	(-0.025-0.049)	(-0.016-0.043)	(-0.023-0.061)		(-0.011-0.016)	(-0.012-0.013)	(-0.007-0.026)	(-0.012-0.015)	(-0.009-0.020)
ML	(-0.040-0.075)	(-0.029-0.040)	(-0.030-0.076)	(-0.043-0.075)	(-0.050-0.090)	(-0.037-0.060)	(-0.039-0.051)	(-0.041-0.059)	(-0.043-0.066)	(-0.102-0.219)	(-0.047-0.067)	(-0.040-0.055)	(-0.034-0.047)	(-0.043-0.063)	(-0.148-0.205)	(-0.033-0.052)	(-0.029-0.049)	(-0.038-0.063)	(-0.032-0.045)	(-0.029-0.053)	(-0.046-0.056)	(-0.03-0.058)		(-0.014-0.013)	(-0.008-0.021)	(-0.012-0.013)	(-0.012-0.021)
TT	(-0.031-0.044)	(-0.022-0.038)	(-0.025-0.059)	(-0.037-0.051)	(-0.035-0.073)	(-0.037-0.039)	(-0.031-0.038)	(-0.032-0.036)	(-0.031-0.038)	(-0.082-0.200)	(-0.036-0.056)	(-0.038-0.048)	(-0.027-0.055)	(-0.028-0.049)	(-0.144-0.209)	(-0.022-0.041)	(-0.022-0.035)	(-0.031-0.051)	(-0.034-0.045)	(-0.020-0.053)	(-0.031-0.052)	(-0.022-0.036)	(-0.035-0.042)		(-0.009-0.019)	(-0.013-0.007)	(-0.015-0.012)
BJ	(-0.027-0.054)	(-0.022-0.042)	(-0.038-0.067)	(-0.022-0.073)	(-0.041-0.077)	(-0.026-0.066)	(-0.026-0.069)	(-0.026-0.054)	(-0.028-0.058)	(-0.087-0.186)	(-0.045-0.075)	(-0.031-0.056)	(-0.028-0.061)	(-0.037-0.057)	(-0.156-0.255)	(-0.039-0.069)	(-0.029-0.062)	(-0.034-0.077)	(-0.034-0.054)	(-0.024-0.068)	(-0.030-0.055)	(-0.019-0.068)	(-0.026-0.072)	(-0.023-0.059)		(-0.013-0.017)	(-0.008-0.026)
LB	(-0.024-0.042)	(-0.022-0.039)	(-0.048-0.038)	(-0.024-0.069)	(-0.047-0.075)	(-0.036-0.034)	(-0.028-0.041)	(-0.029-0.046)	(-0.031-0.047)	(-0.094-0.227)	(-0.049-0.036)	(-0.032-0.056)	(-0.030-0.058)	(-0.039-0.027)	(-0.136-0.228)	(-0.048-0.028)	(-0.023-0.039)	(-0.028-0.051)	(-0.035-0.066)	(-0.029-0.046)	(-0.036-0.047)	(-0.025-0.043)	(-0.033-0.053)	(-0.020-0.033)	(-0.039-0.056)		(-0.013-0.014)
DK	(-0.029-0.054)	(-0.025-0.045)	(-0.024-0.052)	(-0.022-0.046)	(-0.038-0.076)	(-0.029-0.038)	(-0.033-0.040)	(-0.035-0.038)	(-0.034-0.045)	(-0.082-0.198)	(-0.043-0.047)	(-0.036-0.058)	(-0.022-0.096)	(-0.037-0.056)	(-0.127-0.223)	(-0.032-0.065)	(-0.020-0.033)	(-0.037-0.057)	(-0.038-0.055)	(-0.032-0.049)	(-0.035-0.046)	(-0.019-0.051)	(-0.041-0.060)	(-0.025-0.041)	(-0.018-0.067)	(-0.028-0.044)	

Table S3 - Mean validation scores obtained using each of the distinct techniques (GLM, GBM, GAM, FDA, MARS and RF).

Model	AUC mean (±SD)	ROC sensitivity	ROC specificity	TSS mean (±SD)	Sensitivity (±SD)	Specificity (±SD)
GLM	0.901 ±0.022	86.182 ± 7.892	83.694 ± 6.395	0.689 ± 0.049	83.939 ± 7.579	84.792 ± 6.078
GBM	0.931 ±0.021	87.515 ± 5.829	87.745 ± 4.851	0.744 ± 0.059	85.757 ± 6.879	88.451 ± 4.338
GAM	0.903 ±0.025	89.758 ± 6.119	81.370 ± 4.660	0.699 ± 0.049	88.545 ± 6.429	81.311 ± 5.471
FDA	0.879 ±0.030	78.727 ± 7.231	87.260 ± 6.424	0.652 ± 0.064	76.303 ± 6.864	88.502 ± 4.241
MARS	0.879 ±0.099	82.121 ± 15.049	84.213 ± 7.620	0.657 ± 0.112	80.182 ± 14.632	85.311 ± 7.167
RF	0.939 ±0.018	92.424 ± 5.095	86.026 ± 4.060	0.775 ± 0.044	90.848 ± 4.762	86.417 ± 3.823

Table S4 - One-way ANOVA on the effect of minimum sea surface temperature (SST min) and surface air temperature (SAT min) on *P. perna* presence/absence along the warm temperate and cool temperate southwest South African provinces at three trials (Trial 1, Trial 2 and Trial 3). Statistical significant values are depicted in bold.

	Trial 1				Trial 2				Trial 3			
Source	df	MS	F-ratio	P-value	df	MS	F-ratio	P-value	df	MS	F-ratio	P-value
SST min												
Record	1	29.668	28.147	**0.000071**	1	35.149	31.860	**0.000037**	1	46.263	46.658	**0.000004**
Error	16	1.054			16	1.103			16	0.992		
SAT min												
Record	1	0.0775	0.0709	0.796	1	0.722	0.4607	0.511	1	6.5342	3.9276	0.063
Error	16	1.0929			16	1.5672			16	1.6637		

Table S5 – Atlantic and Mediterranean surveyed locations along Iberian shores. Presence of *Mytilus galloprovincialis* depicts potential suitable locations for *P. perna.*

Country	Location	Coordinates	*M. galloprovincialis*
Portugal	Viana do Castelo	41°41’57.85”N; 08°51’23.81”W	Present
Portugal	Ericeira	38°57’21.03”N; 09°25’00.97”W	Present
Portugal	Lizandro	38°56’28.13”N; 09°25’02.28”W	Present
Portugal	Malhão	37°46’44.64”N; 08°48’09.35”W	Present
Portugal	Vila Nova de Milfontes	37°43’04.79”N; 08°47’27.73”W	Present
Portugal	Amoreira	37°20’59.18”N; 08°50’53.15”W	Present
Portugal	Castelejo	37°06’08.00”N; 08°56’44.29”W	Present
Portugal	Sagres	37°00’23.69”N; 08°56’55.80”W	Present
Portugal	Lagos	37°05’55.10”N; 08°40’02.38”W	Present
Portugal	Alvor	37°07’44.45”N; 08°35’47.71”W	Present
Portugal	Pintadinho	37°06’32.07”N; 08°31’11.63”W	Present
Portugal	Albufeira	37°05’21.03”N; 08°11’29.76”W	Present

Portugal	Vilamoura	37°04’05.50”N; 08°06’42.64”W	Present
Portugal	Farol	36°58’29.95”N; 07°51’42.17”W	Present
Portugal	Tavira	37°06’41.06”N; 07°36’57.99”W	Present
Portugal	V. R. de Santo	37°10’03.78”N; 07°24’09.03”W	Present
Spain	Punta del Moral	37°10’57.76”N; 07°19’46.83”W	Present
Spain	Punta Umbria	37°09’53.78”N; 06°56’54.38”W	Present
Spain	Huelva	37°07’45.19”N; 06°51’09.43”W	Absent
Spain	Rota	36°36’55.50”N; 06°21’27.85”W	Absent
Spain	Puerto Sherry	36°34’48.65”N; 06°15’55.07”W	Absent
Spain	Barbate	36°11’00.22”N; 05°56’10.82”W	Absent
Spain	Atlanterra	36°05’24.78”N; 05°48’43.02”W	Present
Spain	Paloma Baja	36°03’49.70”N; 05°43’39.89”W	Absent
Spain	Tarifa	36°00’27.89”N; 05°36’26.09”W	Absent
Spain	Los Palmones	36°10’34.28”N; 05°25’28.26”W	Present
Spain	Torreguadiaro	36°18’00.14”N; 05°16’04.77”W	Present
Spain	La Araña	36°42’41.35”N; 04°19’37.50”W	Present
Spain	Almuñecar	36°43’39.84”N; 03°41’39.86”W	Present
Spain	Balerma	36°41’56.20”N; 02°51’33.99”W	Present
Spain	Roquetas de Mar	36°45’27.41”N; 02°36’15.26”W	Absent
Spain	Aguadulce	36°48’40.91”N; 02°34’00.66”W	Absent
Spain	Cabo de Gata	36°44’00.26”N; 02°12’14.70”W	Present
Spain	La Isleta	36°47’53.54”N; 02°03’42.64”W	Absent
Spain	Garrucha	37°10’21.02”N; 01°49’18.30”W	Absent
Spain	Villaricos	37°14’49.74”N; 01°46’15.80”W	Absent
Spain	Aguilas	37°24’15.16”N; 01°33’56.64”W	Absent
Spain	Calabardina	37°25’53.29”N; 01°30’27.11”W	Absent
Spain	Cape Cope	37°26’12.49”N; 01°29’02.88”W	Absent
Spain	Puerto de Mazarron	37°33’28.51”N; 01°17’10.24”W	Absent
Spain	Portman	37°34’50.35”N; 00°51’13.23”W	Absent
Spain	Cape Palos	37°38’00.94”N; 00°41’23.95”W	Absent
Spain	Guardamar del	38°06’37.33”N; 00°38’27.07”W	Absent
Spain	Santa Pola	38°11’18.94”N; 00°33’06.45”W	Absent
Spain	Alicante	38°21’07.64”N; 00°24’40.40”W	Absent
Spain	Calpe	38°38’25.71”N; 00°03’56.53”E	Absent
Spain	Cape de la Nau	38°43’51.27”N; 00°13’08.85”E	Absent
Spain	Dénia	38°57’50.66”N; 00°07’43.58”W	Present
Spain	Cullera	39°11’16.26”N; 00°13’17.20”W	Present

Table S6 – List of sampling sites for genetic samples of *P. perna* populations.

Country/Territory	Location	Code	Coordinates
Tunisia	Korbous	KR	36°49’29.04”N; 10°33’59.97”E
Tunisia	Bizerte	BZ	37°15’10.41”N; 09°56’41.15”E
Algeria	Annaba bay	AN	36°50’32.48”N; 07°51’17.48”E
Morocco	Punta Negri	PN	35º16’46.33”N; 03º08’06.63”W
Spain	Cape Gata	CG	36º44’00.26”N; 02º12’14.70”W
Spain	Balerma	BM	36º41’56.20”N; 02º51’33.99”W
Spain	Almuñecar	AM	36º43’39.84”N; 03º41’39.86”W
Spain	La Araña	LA	36º42’41.35”N; 04º19’37.50”W
Spain	Torreguadiaro	TG	36º18’00.14”N; 05º16’04.77”W
Spain	Los Palmones	LP	36º10’34.28”N; 05º25’28.26”W
Spain	Atlanterra	AT	36º05’24.78”N; 05º48’43.02”W
Spain	Punta del Moral	PM	37º10’57.76”N; 07º19’46.83”W
Portugal	Tavira	TV	37º06’41.06”N; 07º36’57.99”W
Portugal	Vilamoura	VL	37º04’05.50”N; 08º06’42.64”W
Portugal	Sagres	SG	37º00’23.69”N; 08º56’55.80”W
Morocco	Larache	LR	35°11’48.14”N; 06°09’30.61”W
Morocco	Rabat	RB	34°01’57.26”N; 06°50’27.96"W
Morocco	Casablanca	CB	33°39’07.22”N; 07°29’03.11"W
Morocco	Sidi Bouzid	SB	33°13’06.11”N; 08°34’23.19"W
Morocco	El Beddouza	EB	32°32’42.33”N; 09°16’55.34"W
Morocco	Essaouira	ES	31°30’42.78”N; 09°46’24.31"W
Morocco	Imsouane	IM	30°50’24.43”N; 09°49’21.92"W
Morocco	Mirleft	ML	29°35’06.58”N; 10°02’50.78"W
Morocco	Tan Tan plage	TT	28°30’05.58”N; 11°20’06.38"W
Western Sahara	Boujdour	BJ	26º07’38.95”N; 14º30’02.38’’W
Western Sahara	Nouifed	LB	24º54’30.29”N; 14º49’45.36’’W
Western Sahara	Dakhla	DK	23º46’06.97”N; 15º55’32.16’’W

Table S7 – Presence/absence records of *P. perna* used for the environmental niche modelling approach.

Country	Location	Coordinates	Presence	Reference
Tunisia	Zembra Island	37°08'25.96"N; 10°48'28.94"E	Present	1
Tunisia	Korbous	36°49'29.04"N; 10°33'59.97"E	Present	This study
Tunisia	La Goulette	36°49'18.84"N; 10°18'42.14"E	Present	1
Tunisia	Bizerte	37°15'10.41"N; 09°56'41.15"E	Present	This study
Tunisia	Tabarka	36°57'39.05"N; 08°45'02.62"E	Present	1
Algeria	La Kyenne Beach	36°58'02.00"N; 07°46'24.00"E	Present	2
Algeria	Saint Cloud Beach	36°55'22.20"N; 07°45'50.20"E	Present	2

Algeria	Sunrise Beach	36°54'33.90"N; 07°46'20.90"E	Present	2
Algeria	Sidi Salem Beach	36°52'02.00"N; 07°46'26.60"E	Present	2
Algeria	Annaba Bay	36°50'32.48"N; 07°51'17.48"E	Present	This study
Algeria	Boubouaou El Bahri	36°46'33.56"N; 03°23'15.41"E	Present	3
Algeria	Bordj El Kiffan	36°45'37.72"N; 03°12'45.24"E	Present	3
Spain	Cullera	39°11'16.26"N; 00°13'17.20"W	Absent	This study
Spain	Dénia	38°57'50.66"N; 00°07'43.58"W	Absent	This study
Spain	Cape de la Nau	38°43'51.27"N; 00°13'08.85"E	Absent	This study
Spain	Calpe	38°38'25.71"N; 00°03'56.53"E	Absent	This study
Spain	Alicante	38°21'07.64"N; 00°24'40.40"W	Absent	This study
Spain	Santa Pola	38°11'18.94"N; 00°33'06.45"W	Absent	This study
Spain	Guardamar del Segura	38°06'37.33"N; 00°38'27.07"W	Absent	This study
Spain	Cape Palos	37°38'00.94"N; 00°41'23.95"W	Absent	This study
Spain	Portman	37°34'50.35"N; 00°51'13.23"W	Absent	This study
Spain	Puerto de Mazarron	37°33'28.51"N; 01°17'10.24"W	Absent	This study
Spain	Cape Cope	37°26'12.49"N; 01°29'02.88"W	Absent	This study
Spain	Calabardina	37°25'53.29"N; 01°30'27.11"W	Absent	This study
Spain	Aguilas (beach)	37°24'15.16"N; 01°33'56.64"W	Absent	This study
Spain	Villaricos	37°14'49.74"N; 01°46'15.80"W	Absent	This study
Spain	Garrucha	37°10'21.02"N; 01°49'18.30"W	Absent	This study
Spain	La Isleta (village)	36°48'47.17"N; 02°03'05.46"W	Absent	This study
Spain	La Isleta (beach)	36°47'53.54"N; 02°03'42.64"W	Absent	This study
Spain	Cape Gata	36°44'00.26"N; 02°12'14.70"W	Present	This study
Spain	Aguadulce	36°48'40.91"N; 02°34'00.66"W	Absent	This study
Spain	Roquetas de Mar	36°45'27.41"N; 02°36'15.26"W	Absent	This study
Spain	Balerma	36°41'56.20"N; 02°51'33.99"W	Present	This study
Spain	Almuñecar	36°43'39.84"N; 03°41'39.86"W	Present	This study
Spain	La Araña	36°42'41.35"N; 04°19'37.50"W	Present	This study
Spain	Torreguadiaro	36°18'00.14"N; 05°16'04.77"W	Present	This study
Spain	Los Palmones	36°10'34.28"N; 05°25'28.26"W	Present	This study
Spain	Tarifa	36°00'27.89"N; 05°36'26.09"W	Absent	This study
Spain	Paloma Baja	36°03'49.70"N; 05°43'39.89"W	Absent	This study
Spain	Atlanterra	36°05'24.78"N; 05°48'43.02"W	Present	This study
Spain	Barbate	36°11'00.22"N; 05°56'10.82"W	Absent	This study
Spain	Puerto Sherry	36°34'48.65"N; 06°15'55.07"W	Absent	This study
Spain	Rota	36°36'55.50"N; 06º21'27.85"W	Absent	This study
Spain	Huelva	37°07'45.19"N; 06º51'09.43"W	Absent	This study
Spain	Punta Umbria	37°09'53.78"N; 06º56'54.38"W	Absent	This study
Spain	Punta del Moral	37°10'57.76"N; 07º19'46.83"W	Present	This study
Portugal	Vila Real de Santo António	37°10'03.78"N; 07º24'09.03"W	Present	This study
Portugal	Tavira	37°06'41.06"N; 07º36'57.99"W	Present	This study
Portugal	Farol	36°58'29.95"N; 07º51'42.17"W	Present	This study

Portugal	Vilamoura	37°04'05.50"N; 08º06'42.64"W	Present	This study
Portugal	Albufeira	37°05'21.03"N; 08°11'29.76"W	Absent	This study
Portugal	Pintadinho	37°06'32.07"N; 08°31'11.63"W	Absent	This study
Portugal	Lagos	37°06'01.04"N; 08°40'02.80"W	Absent	This study
Portugal	Sagres	37°00'23.69"N; 08º56'55.80"W	Present	This study
Portugal	Castelejo	37°06'08.00"N; 08º56'44.29"W	Present	This study
Portugal	Amoreira	37°20'59.18"N; 08º50'53.15"W	Absent	This study
Portugal	Vila Nova de Milfontes	37°43'04.79"N; 08º47'27.73"W	Absent	This study
Portugal	Malhão	37°46'44.64"N; 08º48'09.35"W	Absent	This study
Portugal	Lizandro	38°56'28.13"N; 09°25'02.28"W	Absent	This study
Portugal	Ericeira	38°57'21.03"N; 09°25'00.97"W	Absent	This study
Portugal	Viana do Castelo	41°41'57.85"N; 08°51'23.81"W	Absent	This study
Morocco	Punta Negri	35°16'46.33"N; 03º08'06.63"W	Present	This study
Morocco	Larache	35°11'48.14"N; 06°09'30.61"W	Present	This study
Morocco	Bouknadel	34°07'40.74"N; 06°45'14.63"W	Present	4
Morocco	Rabat	34°01'57.26"N; 06°50'27.96"W	Present	This study
Morocco	Skhirat	33°52'15.84"N; 07°03'54.72"W	Present	5
Morocco	Mohammedia	33°42'13.04"N; 07°24'49.62"W	Present	4
Morocco	Casablanca	33°39'07.22"N; 07°29'03.11"W	Present	This study
Morocco	Sidi Bouzid	33°13'06.11"N; 08°34'23.19"W	Present	This study
Morocco	El Beddouza	32°32'42.33"N; 09°16'55.34"W	Present	This study
Morocco	Essaouira	31°30'42.78"N; 09°46'24.31"W	Present	This study
Morocco	Imsouane	30°50'24.43"N; 09°49'21.92"W	Present	This study
Morocco	Anza	30°27'00.18"N; 09°39'44.47"W	Present	This study
Morocco	Mirleft	29°35'06.58"N; 10°02'50.78"W	Present	This study
Morocco	Tan-Tan	28°30'05.58"N; 11°20'06.38"W	Present	This study
Morocco	Tarfaya	27°45'33.64"N; 13°02'40.40"W	Absent	This study
Western Sahara	Boujdour	26°07'38.95"N; 14º30'02.38"W	Present	This study
Western Sahara	Nouifed	24°54'30.29"N; 14º49'45.36"W	Present	This study
Western Sahara	Hassi El kraa	24°41'06.18"N; 14°54'08.87"W	Present	This study
Western Sahara	Dakhla	23°46'06.97"N; 15º55'32.16"W	Present	This study
Mauritania	Cansado – IMROP	20°51'27.19"N; 17°01'52.10"W	Present	6
Mauritania	Cansado – COMECA	20°50'19.68"N; 17°02'01.57"W	Present	6
Mauritania	Cansado – Oil port	20°49'54.71"N; 17°02'07.66"W	Present	6
Mauritania	Nouadhibou	20°58'27.91"N; 17°00'32.18"W	Present	7
Mauritania	Nouakchott	18°05'09.87"N; 16°01'35.15"W	Present	7
Senegal	Dakar	14°40'47.39"N; 17°28'11.23"W	Present	8
Ivory Coast	Ebrie Lagoon	05°17'36.29"N; 04°00'37.57"W	Present	9
Ghana	Benya lagoon	05°04'57.21"N; 01°20'48.94"W	Present	10

Ghana	Sakumo lagoon	05°31'02.85"N; 00°16'52.32"W	Present	10
Congo	Pointe-Noire	04°45'32.64"S; 11°50'13.97"E	Present	11
Angola	Luanda	08°52'11.28"S; 13°11'42.93"E	Present	This study
Namibia	Rocky Point	18°59'36.13"S; 12°28'36.54"E	Present	This study
Namibia	Mowey Bay	19°22'22.61"S; 12°42'19.38"E	Present	This study
Namibia	Terrace Bay	19°59'49.09"S; 13°02'01.24"E	Present	This study
Namibia	Swakopmound	22°40'07.52"S; 14°31'23.08"E	Present	This study
Namibia	Walvis Bay	22°55'28.69"S 14°31'03.66"E	Present	This study
Namibia	Sylvia Hill	25°08'46.62"S; 14°50'43.44"E	Present	This study
Namibia	Luderitz	26°38'43.31"S; 15°05'25.62"E	Absent	This study
South Africa	Alexander Bay	28°40'05.74"S; 16°30'20.35"E	Absent	This study
South Africa	Port Nolloth	29°15'01.53"S; 16°52'02.30"E	Absent	This study
South Africa	Brand-se-Baai	31°17'30.09"S; 17°52'46.86"E	Absent	This study
South Africa	Paternoster	32°48'07.22"S 17°53'51.95"E	Absent	This study
South Africa	Elands Bay	32°18'59.49"S; 18°18'54.50"E	Absent	This study
South Africa	Saldanha Bay	33°01'34.14"S; 17°56'30.55"E	Absent	This study
South Africa	Yzerfontein	33°21'14.99"S; 18°09'01.07"E	Absent	This study
South Africa	Bloubergstrand	33°48'06.18"S; 18°27'30.45"E	Absent	This study
South Africa	Camps Bay	33°57'11.26"S; 18°22'27.61"E	Absent	This study
South Africa	Scarborough	34°11'55.95"S; 18°22'16.49"E	Present	This study
South Africa	Outland Point	34°14'20.82"S; 18°28'38.02"E	Present	This study
South Africa	Pringle Bay	34°20'16.52"S; 18°49'41.04"E	Present	This study
South Africa	Betty's Bay	34°21'23.82"S; 18°55'59.54"E	Present	This study
South Africa	Kleinmond	34°20'37.84"S; 19°02'12.05"E	Present	This study
South Africa	Hermanus	34°24'40.64"S; 19°15'40.67"E	Present	This study
South Africa	Gans Bay	34°33'17.92"S; 19°21'49.38"E	Present	This study
South Africa	Cape Aghullas	34°49'26.40"S; 20°01'01.59"E	Present	This study
South Africa	Arniston	34°40'08.12"S; 20°14'05.13"E	Present	This study
South Africa	Mossel Bay-lighthouse	34°10'58.42"S; 22°09'30.73"E	Present	This study
South Africa	Mossel Bay-Dias strand	34°10'27.36"S; 22°08'09.70"E	Present	This study
South Africa	Mossel Bay-Hartenbos	34°09'56.22"S; 22°06'51.08"E	Present	This study
South Africa	Glentana	34°03'07.41"S; 22°19'19.01"E	Present	This study
South Africa	Wilderness	33°59'49.68"S; 22°33'57.53"E	Present	This study
South Africa	Sedgefield	34°01'44.56"S; 22°46'05.08"E	Present	This study
South Africa	Brenton-on-Sea	34°04'28.83"S; 23°01'12.56"E	Present	This study
South Africa	Robberg-open	34°06'08.06"S; 23°22'52.92"E	Present	This study
South Africa	Robberg-in	34°05'57.04"S; 23°22'37.86"E	Present	This study
South Africa	Plettenberg Bay-Beacon Isle	34°03'35.65"S; 23°22'50.22"E	Present	This study
South Africa	Plettenberg Bay-Lookout	34°03'06.08"S; 23°22'39.02"E	Present	This study
South Africa	Pletternberg Bay-Keurboomstrand	34°00'17.78"S; 23°27'18.61"E	Present	This study
South Africa	Oubosstrand	34°04'22.88"S; 24°13'29.60"E	Present	This study

South Africa	Seal Point	34°12'35.27"S; 24°49'31.96"E	Present	This study
South Africa	Sea Vista	34°10'15.24"S; 24°50'05.23"E	Present	This study
South Africa	Jeffrey's Bay	34°03'37.00"S; 24°55'43.64"E	Present	This study
South Africa	Noordkloofspunt	34°01'34.07"S; 24°55'50.90"E	Present	This study
South Africa	Kini Bay	34°01'17.91"S; 25°22'48.39"E	Present	This study
South Africa	Skoenmakerskop	34°02'28.06"S; 25°32'01.70"E	Present	This study
South Africa	Chelsea Point	34°02'45.66"S; 25°38'04.55"E	Present	This study
South Africa	Port Elizabeth-Bird Rock	33°59'00.57"S; 25°40'17.17"E	Present	This study
South Africa	Port Elizabeth-Shark Rock	33°58'47.18"S; 25°39'28.38"E	Present	This study
South Africa	Port Elizabeth-Deal Park	33°52'54.56"S; 25°37'37.39"E	Present	This study
South Africa	Hougham Park	33°45'17.09"S; 25°46'01.20"E	Present	This study
South Africa	Kenton-on-Sea	33°41'37.49"S; 26°40'13.70"E	Present	This study
South Africa	Old Womans	33°28'56.45"S; 27°09'06.98"E	Present	This study
South Africa	Kayser's Beach	33°12'44.21"S; 27°36'41.05"E	Present	This study
South Africa	Kidd's Beach	33°08'49.12"S; 27°42'11.29"E	Present	This study
South Africa	Gonubie	32°56'18.43"S; 28°01'58.06"E	Present	This study
South Africa	Glen Muir	32°53'04.40"S; 28°06'00.23"E	Present	This study
South Africa	Haga Haga	32°45'54.29"S; 28°14'56.51"E	Present	This study
South Africa	Morgan Bay	32°42'36.11"S; 28°20'30.00"E	Present	This study
South Africa	Kei mounth	32°41'03.70"S; 28°22'56.63"E	Present	This study
South Africa	Port St Johns	31°39'15.23"S; 29°30'56.40"E	Present	This study
South Africa	Port Edward	31°03'15.39"S; 30°13'42.30"E	Present	This study
South Africa	Durban	29°51'10.77"S; 31°02'27.68"E	Present	This study
South Africa	Ballito	29°32'42.96"S; 31°12'57.11"E	Present	This study
South Africa	Mapelane	28°24'27.04"S; 32°25'37.35"E	Present	This study
South Africa	Kosi Bay	27°00'45.82"S; 32°52'02.40"E	Present	This study
South Africa	Marion Island	46°49'57.95"S; 37°47'13.61"E	Absent	This study
Mozambique	Punta Douro	26°50'39.15"S; 32°53'41.39"E	Present	This study
Mozambique	Praia de Xai-Xai	25°06'42.09"S; 33°45'17.90"E	Present	This study
Mozambique	Praia de Závora	24°31'15.10"S; 35°12'22.40"E	Present	This study
Mozambique	Baía de Inhambane	23°52'04.68"S; 35°22'47.26"E	Absent	This study
Mozambique	Chicamane	21°53'09.91"S; 35°18'18.13"E	Absent	This study
Mozambique	Macunhe	21°50'30.97"S; 35°17'43.32"E	Absent	This study
Mozambique	Nhagonzo	21°43'50.44"S; 35°17'01.16"E	Absent	This study
Mozambique	Bazaruto	21°32'25.26"S; 35°28'14.60"E	Absent	This study
Yemen	Ash-shehr	14°44'49.88"N; 49°35'31.46"E	Present	12
Yemen	Bandar Fuqum	12°44'52.37"N; 44°49'31.63"E	Present	13
Yemen	Ras Marbat	12°47'08.56"N; 44°58'17.13"E	Present	13
Yemen	Sira Island	12°46'37.04"N; 45°02'55.42"E	Present	13
Oman	Salalah - Raysut	16°59'49.12"N; 54°05'23.18"E	Present	14
Oman	Muscat	23°37'19.37"N; 58°35'22.14"E	Present	15

Table S8 – Environmental variables used to model *P. perna* likelihood of presence. SST indicates sea surface temperature. SAT indicates surface air temperature.

	Study area		Presences	
Predictors (units)	Min-Max	Mean	Min-Max	Mean
Cloud cover	0.069-0.943	0.531	0.251-0.872	0.49
Waves (m)	0.388-5.774	2.008	0.625-3.829	2.183
Minimum SST (^{o}C)	-1.585-28.827	17.403	13.817-25.977	17.316
Maximum SST	6.979-37.600	27.335	18.908-31.705	24.028
Minimum SAT (^{o}C)	-7.757-25.477	12.414	5.925-23.069	13.878
Maximum SAT	8.298-35.289	28.169	22.055-34.562	26.511
Salinity	1.634-41.475	34.66	33.953-37.277	35.827
Nitrate (µmol/L)	0.003-12.494	1.355	0.548-6.695	2.12
Phosphate	0.0184-	0.229	0.049-0.995	0.33

Table S9 – Microsatellite data of 732 individuals of *P. perna* used in nuclear genetic analyses. POP refers to populations as in Supplementary Table S6. The 0 value depicted null alleles.

	P08		P16		P20		P26		P01		P05		P27		P02	
POP	Allele A	Allele B	Allele A	Allele B	Allele A	Allele B	Allel e A	Allele B	Allele A	Allele B	Allele A	Allele B	Allele A	Allele B	Allele A	Allele B
KR	111	111	103	172	213	213	100	109	157	157	130	132	108	140	112	112
KR	111	126	0	0	207	326	100	104	165	167	127	132	108	142	112	116
KR	115	115	98	103	330	356	94	98	165	167	119	127	136	153	112	112
KR	111	115	103	103	202	202	92	100	169	169	115	125	108	138	112	112
KR	111	119	107	122	353	353	100	102	165	167	109	119	83	142	112	112
KR	115	115	105	105	209	215	100	107	166	167	115	117	144	174	112	120
KR	111	115	103	132	215	215	96	98	175	175	123	130	108	146	112	112
KR	115	119	103	103	351	360	98	104	167	167	121	123	103	178	112	112
KR	119	126	153	153	213	311	100	102	158	165	123	123	140	142	112	280
KR	111	126	103	105	207	317	98	100	167	167	121	123	140	142	112	112
KR	111	119	103	103	202	315	98	102	167	167	115	117	108	138	112	220
KR	111	119	103	103	338	338	102	106	160	167	119	136	108	138	112	112
KR	115	126	115	115	200	202	102	104	157	157	119	125	108	165	112	112
KR	115	119	103	113	230	344	102	115	157	167	132	132	108	153	112	290
KR	111	115	103	103	206	334	94	98	169	177	119	121	108	140	112	112
KR	111	115	103	113	207	310	104	104	157	165	115	130	108	108	112	116
KR	111	115	103	126	202	226	92	107	171	173	121	130	140	174	112	114
KR	115	115	105	105	300	355	102	236	173	173	113	119	110	144	112	112
KR	111	115	122	122	212	356	102	109	165	165	119	134	140	151	112	112
KR	115	115	98	98	221	295	102	102	167	167	115	115	108	142	112	112
KR	111	115	105	105	358	358	102	166	165	169	123	130	113	142	106	112
KR	111	115	115	115	249	353	94	208	162	171	119	134	146	159	112	112

KR	111	119	103	103	221	221	96	102	167	173	115	117	140	140	89	110
KR	111	119	105	118	204	215	104	107	160	167	107	119	108	174	0	0
KR	111	115	105	113	291	338	100	102	171	185	119	134	108	134	112	112
KR	111	119	103	103	308	334	104	107	165	167	125	150	108	138	0	0
KR	111	115	109	109	226	353	98	100	157	169	113	127	108	108	112	259
KR	111	111	109	134	309	309	102	104	167	167	115	115	138	140	112	417
KR	111	111	103	126	206	317	94	102	167	169	115	115	146	153	112	112
KR	111	119	126	126	321	338	102	104	165	165	117	119	108	140	112	112
BZ	115	119	140	140	332	332	102	107	165	165	115	119	138	153	110	112
BZ	111	115	103	103	234	370	96	104	165	165	115	123	108	140	112	135
BZ	111	126	103	105	202	217	100	100	167	167	119	119	140	202	112	112
BZ	107	115	103	103	202	368	102	102	165	175	115	127	108	151	112	112
BZ	115	126	103	103	326	368	102	104	167	167	117	125	107	144	110	112
BZ	115	119	102	102	326	364	100	106	171	171	130	138	108	108	89	112
BZ	111	115	103	126	206	375	100	102	165	167	115	121	108	140	112	112
BZ	111	115	0	0	206	347	100	106	157	167	117	130	110	140	112	112
BZ	111	111	103	120	305	379	102	106	157	162	130	134	108	108	112	131
BZ	111	115	103	103	192	383	102	102	167	177	115	121	140	144	110	112
BZ	115	115	105	120	332	332	98	197	165	165	115	136	138	138	112	251
BZ	115	115	128	128	353	364	106	109	169	173	123	125	138	163	112	112
BZ	111	119	124	124	207	364	96	100	157	157	123	125	108	138	112	112
BZ	111	111	98	111	202	313	92	104	167	167	115	132	108	108	112	112
BZ	111	115	103	103	202	351	98	102	165	165	119	125	163	170	112	290
BZ	111	115	103	103	207	347	98	104	165	165	119	123	140	142	112	112
BZ	115	115	100	105	207	246	98	104	165	165	117	140	144	149	112	131
BZ	111	119	126	126	209	362	100	100	165	171	115	117	138	146	112	112
BZ	111	111	103	103	225	315	98	102	165	165	123	138	138	149	112	118
BZ	107	115	103	103	226	362	102	102	167	167	119	127	138	140	112	290
BZ	119	119	260	260	283	360	98	106	167	167	115	115	140	140	0	0
BZ	115	119	105	105	215	344	100	104	165	165	119	152	108	146	112	112
BZ	111	111	98	103	370	371	102	104	169	171	117	119	108	138	112	273
BZ	111	111	103	128	206	207	98	98	165	169	130	130	108	136	112	112
BZ	111	111	107	107	0	0	102	106	167	175	119	130	108	108	112	245
BZ	111	111	102	102	234	358	100	100	158	167	113	134	108	187	112	112
BZ	111	111	128	128	334	336	100	102	165	173	115	136	110	138	112	112
BZ	111	115	103	103	295	295	107	109	165	169	115	117	108	144	112	112
BZ	111	115	102	126	190	307	94	98	171	171	115	121	140	153	112	112
BZ	111	115	105	124	332	371	100	102	159	167	119	127	108	151	112	255
BZ	111	111	103	103	287	356	98	107	165	167	123	125	139	139	112	240
BZ	111	115	0	0	213	345	102	106	165	167	119	123	108	140	89	110
OR	111	115	103	103	200	206	98	106	165	167	119	121	108	108	0	0
OR	111	111	103	103	200	321	102	102	155	167	115	115	108	163	112	114
OR	115	115	107	115	204	209	92	92	167	169	119	123	110	140	0	0
OR	111	115	103	103	308	345	100	104	167	167	123	138	165	193	112	112

OR	111	115	136	136	200	317	98	109	165	167	115	127	108	168	112	261
OR	111	119	0	0	204	209	102	104	165	165	115	132	108	146	112	112
OR	111	111	128	128	307	313	98	104	157	171	115	130	110	138	112	114
OR	111	111	103	103	198	309	98	104	165	167	119	134	110	144	112	269
OR	115	119	144	144	200	379	98	104	165	165	125	125	101	112	112	135
OR	111	111	103	107	213	315	100	104	158	167	123	123	108	144	112	112
OR	111	119	126	126	202	321	98	102	167	171	125	145	108	151	112	112
OR	111	115	103	155	228	345	100	102	165	167	115	115	108	161	112	129
OR	111	111	0	0	328	360	98	100	165	167	119	127	140	142	110	110
OR	111	119	103	103	207	356	102	106	167	171	119	132	108	163	110	112
OR	111	115	115	124	349	349	102	102	165	177	116	121	134	140	0	0
OR	111	119	103	103	307	311	102	104	167	167	117	119	138	146	112	112
OR	111	111	0	0	309	309	90	102	157	165	115	130	108	149	112	112
OR	115	119	136	136	200	338	100	102	157	169	0	0	110	157	112	112
OR	111	115	113	128	209	209	98	104	165	165	119	134	108	142	112	137
OR	111	111	262	262	213	334	106	107	165	167	115	119	108	182	112	118
OR	111	119	103	103	221	330	98	134	167	175	125	132	107	108	0	0
OR	111	111	103	103	190	321	102	109	165	167	127	132	108	136	112	290
PN	115	119	157	157	217	351	94	109	168	171	115	130	108	138	112	131
PN	111	111	0	0	209	218	98	102	167	169	115	115	108	153	112	112
PN	111	111	98	98	215	215	102	106	165	167	115	115	108	138	112	112
PN	111	115	102	103	221	313	94	106	157	171	115	119	144	155	112	112
PN	115	119	98	103	224	234	106	201	167	175	132	169	108	155	112	112
PN	111	123	103	103	299	342	98	294	165	167	127	138	108	128	112	288
PN	119	126	103	103	221	303	102	109	167	167	115	121	140	142	112	112
PN	111	115	103	144	220	321	102	106	167	167	121	125	108	144	112	112
PN	111	123	103	103	192	222	102	102	167	167	123	130	108	157	112	112
PN	111	115	105	107	0	0	107	111	167	167	125	140	138	140	112	112
PN	111	119	103	195	309	344	102	104	167	167	115	134	108	110	112	114
PN	111	115	115	136	225	321	98	100	157	175	127	140	108	108	112	112
PN	111	111	103	103	311	351	94	100	165	165	115	130	108	110	112	257
PN	111	115	103	105	309	360	98	102	165	171	132	143	108	108	112	112
PN	115	126	103	103	340	340	100	104	157	157	113	130	108	142	112	135
PN	111	115	115	115	218	383	102	102	167	167	123	130	159	174	112	135
PN	115	115	103	113	204	207	104	107	165	173	119	132	155	157	112	112
PN	111	115	103	105	217	334	102	104	165	175	121	121	108	108	112	112
PN	111	112	0	0	328	330	98	104	171	181	116	121	149	170	112	112
PN	111	119	103	103	202	383	98	104	165	167	109	115	108	144	112	114
PN	111	115	0	0	287	332	102	104	167	169	125	130	138	138	110	112
PN	111	123	103	105	206	246	102	106	167	167	115	121	108	138	110	112
PN	111	111	103	105	207	330	98	98	165	165	119	123	138	140	110	110
PN	111	119	103	144	206	308	102	109	167	167	115	121	108	108	112	112
PN	115	119	103	128	383	396	98	106	167	169	121	127	108	157	112	257
PN	115	115	103	105	0	0	98	107	155	165	115	125	108	163	112	114

PN	111	111	0	0	192	324	104	106	157	175	119	123	140	140	112	112
PN	111	111	103	103	207	319	102	104	165	169	111	121	108	142	112	112
PN	111	111	103	103	217	315	102	102	163	167	121	132	108	165	112	235
PN	111	111	107	126	320	358	98	106	167	167	109	115	140	155	0	0
PN	111	115	124	124	292	321	102	104	165	167	132	145	108	182	112	112
CG	111	111	203	203	200	209	98	107	165	167	127	134	108	108	112	112
CG	111	119	138	138	345	345	92	94	165	165	115	127	108	138	112	116
CG	111	115	98	98	206	368	104	176	165	173	113	115	140	142	112	253
CG	111	111	0	0	321	345	100	102	165	167	115	119	108	163	89	112
CG	111	115	0	0	356	379	100	104	165	171	121	127	138	161	112	112
CG	111	119	0	0	202	217	92	98	167	171	115	117	144	149	112	112
CG	115	119	103	103	330	337	98	102	165	165	119	125	144	163	112	292
CG	115	130	118	130	299	299	104	104	167	167	109	132	138	140	110	112
CG	111	111	124	124	319	330	98	102	167	167	117	127	136	157	112	112
CG	111	115	120	120	224	332	104	106	165	171	115	119	138	149	112	118
CG	115	119	0	0	206	347	102	102	167	167	115	138	108	108	112	112
CG	111	115	107	107	304	345	102	109	165	170	115	130	108	140	112	112
CG	119	119	142	142	188	383	102	104	160	160	115	119	136	140	112	112
BM	111	126	103	105	321	353	102	109	167	167	125	130	108	161	112	112
BM	111	115	103	122	305	313	98	102	171	171	115	123	107	108	114	114
BM	111	115	105	134	321	321	100	104	165	167	117	123	108	144	112	112
BM	111	115	103	103	206	330	102	201	165	171	119	123	138	140	112	135
BM	111	111	103	103	207	241	109	164	167	167	115	132	108	233	110	112
BM	111	119	103	103	202	379	92	102	165	165	113	119	108	108	112	112
BM	119	119	124	208	336	336	109	109	158	181	123	130	138	140	112	112
BM	111	120	105	105	204	324	102	104	167	167	123	125	140	146	112	112
BM	111	111	103	147	226	313	106	205	167	171	115	136	140	142	112	112
BM	111	111	103	103	202	211	98	100	157	167	123	123	111	140	112	112
BM	111	115	103	103	215	221	100	104	165	165	125	125	108	108	112	112
BM	111	115	0	0	202	345	98	102	165	165	113	119	108	186	112	261
BM	111	126	103	105	226	319	104	106	157	165	119	143	108	108	112	112
BM	111	111	115	115	202	204	102	109	165	169	117	121	165	176	106	112
BM	111	115	103	126	206	237	98	98	155	167	119	125	108	186	112	210
BM	111	115	111	140	206	373	104	109	157	165	113	115	140	151	112	112
BM	111	115	0	0	230	364	98	106	167	171	115	127	142	144	112	112
BM	111	119	0	0	200	336	107	107	165	171	130	140	113	140	112	112
BM	115	119	0	0	202	209	98	113	158	165	123	132	108	155	112	112
BM	111	119	103	103	351	391	100	227	155	157	119	136	112	151	112	112
BM	111	111	107	218	213	316	102	102	159	165	109	134	108	108	112	222
BM	111	123	103	107	0	0	96	102	165	173	117	123	132	136	112	112
BM	111	111	103	103	217	326	94	98	165	167	115	121	161	180	110	112
BM	111	111	103	103	202	338	98	113	157	171	115	127	108	146	112	112
BM	111	115	105	105	321	330	102	104	168	170	119	132	108	140	112	112
BM	111	126	115	128	200	204	102	102	167	167	125	132	140	151	112	112

BM	111	119	113	113	206	338	102	109	167	167	119	167	108	149	112	112
BM	119	119	103	103	230	379	100	102	167	171	115	119	138	155	112	112
BM	111	111	98	128	215	317	98	102	165	167	117	125	138	159	112	139
BM	111	111	103	103	221	355	94	98	165	165	115	134	108	180	112	112
BM	115	115	128	128	207	221	102	106	167	171	130	150	134	134	112	112
BM	111	115	105	105	347	397	102	104	165	167	115	123	138	155	112	235
AM	119	119	103	107	338	338	102	107	165	165	132	134	142	159	112	273
AM	111	115	0	0	204	215	102	106	165	167	123	130	110	138	112	114
AM	111	119	103	103	200	326	98	109	167	167	123	130	108	161	112	112
AM	111	115	103	103	313	318	102	107	165	167	115	115	138	170	112	112
AM	119	119	0	0	206	217	94	100	167	171	115	134	146	149	112	112
AM	111	111	142	142	219	334	102	102	171	171	115	134	108	108	112	112
AM	111	115	103	103	279	307	100	102	167	169	123	130	108	146	106	112
AM	107	111	128	128	202	305	100	102	167	167	115	121	155	186	112	112
AM	111	115	103	105	326	362	106	106	167	167	125	132	108	149	110	112
AM	111	111	113	113	358	364	100	102	165	165	113	119	108	146	112	112
AM	111	111	103	103	353	360	104	113	165	171	119	127	107	140	112	112
AM	111	111	0	0	237	371	98	104	167	167	136	138	108	108	112	112
AM	111	111	262	262	244	244	100	102	167	175	123	125	108	136	112	222
AM	111	115	0	0	289	334	102	104	165	165	130	130	138	155	112	247
AM	115	115	103	151	413	413	102	107	171	173	116	123	128	153	112	112
AM	111	115	103	170	326	349	94	104	165	169	117	119	110	140	112	112
AM	111	111	103	103	221	315	94	102	163	167	121	140	140	144	112	112
AM	115	115	100	103	204	317	102	109	171	171	116	127	140	178	112	114
AM	111	115	103	155	206	347	102	107	167	171	123	125	108	161	112	112
AM	111	111	174	174	334	356	102	106	165	167	117	145	108	180	0	0
AM	111	119	0	0	221	386	98	102	165	173	115	119	155	170	112	112
AM	111	111	103	105	207	340	104	107	167	167	115	117	140	144	0	0
AM	111	126	118	118	287	287	92	104	165	167	116	116	136	140	112	249
AM	111	130	105	113	202	383	98	115	167	175	109	127	108	140	112	112
AM	111	115	103	103	0	0	102	106	165	167	123	127	108	110	112	112
AM	111	115	103	103	209	347	102	102	177	177	119	130	108	140	112	112
AM	111	115	115	115	206	206	98	98	165	177	115	115	138	157	112	112
AM	115	115	103	103	215	342	104	106	167	167	111	125	131	168	112	112
AM	111	111	103	132	204	223	102	107	165	165	115	125	138	138	89	112
AM	115	115	103	124	344	344	104	106	158	158	121	125	110	128	112	245
AM	119	126	103	113	206	309	104	106	165	167	113	127	108	138	112	112
AM	111	115	103	109	202	344	102	106	165	167	127	127	107	138	112	112
AM	111	111	103	103	319	370	104	104	165	167	127	132	138	180	112	112
LA	119	119	103	148	211	317	102	107	165	165	115	125	110	142	112	450
LA	111	119	103	155	206	318	98	104	170	173	123	127	134	140	112	112
LA	111	115	103	103	207	237	96	98	165	167	113	115	108	138	112	112
LA	111	119	103	103	202	207	92	102	165	165	123	127	108	149	112	112
LA	115	115	98	98	315	342	102	104	165	169	109	115	108	110	112	112

LA	115	126	103	103	303	332	98	100	165	165	121	130	108	140	112	112
LA	111	111	103	105	336	347	102	106	167	167	115	125	108	140	112	112
LA	107	111	103	103	213	347	98	102	157	157	127	134	108	108	110	112
LA	111	115	103	103	204	386	102	104	157	167	117	134	108	138	112	131
LA	111	115	115	115	206	349	102	168	158	167	115	130	108	132	112	112
LA	115	119	103	124	383	383	100	102	158	158	123	145	108	111	112	112
LA	111	119	103	103	311	355	100	102	167	177	109	117	108	140	112	112
LA	111	115	103	103	360	371	98	98	167	167	115	119	108	149	112	131
LA	111	111	103	103	206	215	100	102	167	167	115	119	108	134	112	131
LA	111	115	120	120	200	206	106	106	165	167	119	119	142	144	112	112
LA	111	111	128	136	202	230	104	107	160	167	123	130	108	140	112	135
LA	111	111	103	107	206	217	100	109	167	167	123	130	140	140	112	112
LA	111	119	103	103	221	324	92	98	165	167	121	127	142	168	110	114
LA	111	111	105	105	206	300	94	96	165	165	121	125	108	166	89	112
LA	115	115	115	115	306	336	104	160	167	167	119	123	140	165	112	278
LA	111	111	103	122	315	375	94	104	160	167	119	123	136	144	112	112
LA	111	111	103	107	202	207	100	111	157	160	115	123	110	151	112	112
LA	111	111	111	111	251	311	106	212	165	167	115	117	108	140	110	112
LA	111	111	115	115	192	360	102	104	160	165	119	132	132	138	106	106
LA	111	111	126	144	208	215	100	107	169	169	119	127	107	142	112	112
LA	111	115	140	153	351	351	100	109	167	167	115	121	138	163	112	112
LA	111	115	172	172	251	311	98	104	165	171	123	130	136	138	112	112
LA	111	115	102	103	206	362	102	106	157	165	125	132	142	185	112	112
LA	111	115	113	113	190	328	94	102	165	167	121	138	108	108	112	112
LA	111	115	103	103	221	221	92	102	169	171	117	127	110	146	89	110
TG	115	115	0	0	206	225	94	98	165	167	115	138	111	124	0	0
TG	111	111	100	100	206	215	102	106	165	167	119	125	151	153	112	112
TG	111	111	103	117	211	211	98	104	157	165	115	117	134	140	112	112
TG	111	115	0	0	302	334	102	109	157	157	115	119	108	108	112	112
TG	111	115	109	109	318	336	102	102	157	167	127	136	108	108	112	112
TG	111	119	103	103	204	315	98	102	167	167	113	123	110	142	112	112
TG	111	126	103	103	329	329	102	106	157	167	123	127	151	163	112	112
TG	115	115	103	103	0	0	98	104	165	167	145	154	108	108	112	112
TG	111	126	103	103	230	366	106	109	162	165	123	132	138	151	110	112
TG	115	115	103	105	213	226	92	106	167	167	119	125	108	108	112	112
TG	111	111	0	0	200	353	98	100	165	165	134	136	149	180	112	112
TG	111	115	107	107	299	317	104	109	167	167	121	125	110	138	112	112
TG	111	115	103	103	202	303	98	98	157	167	115	119	108	161	112	114
TG	115	119	103	157	321	340	98	106	165	167	119	125	108	142	112	112
TG	111	111	103	109	334	345	98	104	169	169	119	125	112	140	112	112
TG	115	115	98	103	206	321	96	109	167	173	115	125	111	142	112	112
TG	111	115	0	0	309	326	102	106	165	167	115	132	142	163	112	112
TG	115	115	103	148	202	345	107	201	167	167	121	123	108	136	112	112
TG	111	119	157	157	207	340	106	109	165	165	127	132	142	149	112	112

TG	111	115	103	113	211	211	104	104	157	157	115	119	163	199	112	112
TG	115	119	0	0	202	221	102	115	165	167	115	123	110	163	110	112
TG	111	115	0	0	309	347	94	98	171	171	121	123	133	157	112	112
TG	107	111	105	105	200	221	98	102	167	171	119	130	108	127	112	112
TG	115	119	117	117	206	324	98	102	165	167	117	127	108	142	106	112
TG	111	115	105	113	213	311	94	104	157	167	119	134	101	108	112	112
TG	115	119	102	103	202	204	102	102	165	167	125	125	108	140	112	135
TG	111	111	103	113	206	386	98	104	167	169	115	119	142	163	112	112
TG	111	119	103	103	209	349	100	106	169	169	119	136	151	151	114	114
TG	111	119	98	98	221	237	100	102	165	167	115	121	108	174	114	114
TG	111	111	0	0	317	375	94	100	163	165	115	138	144	144	112	112
TG	115	115	103	113	207	254	100	107	165	173	115	116	142	182	112	112
TG	115	115	128	163	283	303	102	109	167	167	115	123	140	140	0	0
TG	111	111	103	103	231	334	102	128	169	171	119	121	138	142	0	0
LP	111	115	103	103	202	347	106	164	167	169	115	152	157	157	112	112
LP	111	115	103	103	190	347	102	104	165	167	119	138	108	108	112	112
AT	115	119	103	132	309	330	94	102	167	171	115	125	108	157	112	112
AT	115	119	103	103	223	308	100	102	167	169	127	130	108	140	112	112
AT	111	115	0	0	0	0	94	109	158	158	119	134	144	155	112	112
AT	111	115	103	103	206	207	107	111	157	165	119	130	108	149	112	112
AT	111	111	105	118	206	217	102	106	167	167	113	121	108	136	110	112
AT	111	111	103	103	231	360	104	106	165	171	125	132	107	142	110	112
AT	111	111	103	103	204	342	98	104	167	171	115	119	108	149	112	112
AT	120	126	0	0	338	380	100	104	167	171	119	152	146	153	110	112
AT	111	111	346	346	207	356	94	104	155	171	116	125	138	140	112	112
AT	123	134	0	0	309	332	98	100	167	171	115	123	134	140	112	112
AT	111	111	103	157	225	353	102	104	163	171	115	125	146	149	112	114
AT	111	119	103	103	226	345	104	203	169	169	109	119	138	144	112	112
AT	111	119	103	103	355	355	98	106	165	167	115	115	108	138	112	114
AT	115	126	113	113	340	397	102	102	160	165	125	127	138	138	112	112
AT	111	119	103	148	313	334	102	102	167	171	115	132	140	144	112	135
AT	115	126	0	0	202	328	98	102	165	165	123	148	140	159	112	203
AT	111	119	105	126	200	206	102	104	163	167	123	130	108	108	112	112
AT	119	119	103	103	246	246	106	109	167	171	121	130	129	184	112	290
AT	111	119	103	103	221	379	102	106	165	165	113	121	108	138	112	278
AT	107	115	103	113	321	321	102	102	158	165	148	148	108	140	112	230
AT	111	111	105	105	192	204	102	102	167	169	115	121	140	151	112	112
AT	111	119	0	0	202	209	92	98	167	171	119	125	136	142	112	112
AT	111	111	105	109	219	287	100	104	165	167	117	127	108	168	112	112
AT	115	119	161	161	206	228	102	106	165	171	134	134	108	110	112	112
AT	111	119	0	0	206	304	98	98	185	185	115	123	149	149	112	114
AT	111	115	98	124	328	353	102	104	157	167	119	127	108	180	112	116
PM	111	111	103	103	347	356	98	102	157	167	130	132	155	166	112	112
PM	111	115	100	103	207	321	102	104	165	165	115	119	138	201	112	129

PM	111	130	103	115	215	296	107	109	159	171	119	132	140	151	112	112
PM	115	115	130	130	313	371	109	172	167	173	109	134	108	176	112	206
PM	115	115	109	109	192	360	98	102	167	168	125	130	108	174	112	112
PM	115	119	103	103	366	375	98	102	158	165	119	148	142	146	112	112
PM	111	111	0	0	0	0	92	102	165	169	115	119	108	142	112	112
PM	111	126	103	103	304	321	94	100	165	167	121	145	144	146	89	112
PM	111	115	103	103	355	364	100	104	165	165	115	127	108	110	112	112
PM	111	134	102	105	344	368	98	100	169	169	123	125	142	146	89	112
PM	115	119	103	103	334	356	109	117	165	165	115	123	108	108	112	112
PM	111	111	107	138	252	324	100	106	158	171	119	119	108	126	112	112
PM	111	115	103	107	206	221	102	104	165	165	119	119	138	146	112	112
PM	111	123	130	130	308	326	98	102	171	171	115	145	108	153	112	245
PM	111	111	0	0	198	228	102	102	167	171	115	130	110	138	112	112
PM	115	115	102	161	215	352	104	107	168	168	115	119	108	140	112	112
PM	111	115	98	98	326	326	92	104	165	165	121	130	138	144	110	110
PM	115	119	155	164	347	347	102	102	165	167	115	143	108	165	0	0
PM	117	126	0	0	221	319	102	102	167	167	132	140	136	142	112	114
PM	111	111	103	103	204	336	98	102	165	165	121	150	108	144	112	112
PM	111	115	0	0	287	287	94	100	165	165	119	121	140	144	112	112
PM	111	119	103	113	202	353	104	109	165	175	134	134	108	191	112	264
TV	111	115	0	0	332	342	102	109	165	165	115	119	140	142	112	112
TV	111	126	103	105	206	246	100	102	167	167	119	140	144	146	112	249
TV	111	119	103	138	209	209	100	208	165	171	123	127	108	110	112	112
TV	111	115	103	109	313	375	100	104	171	173	115	134	108	142	112	112
TV	111	111	0	0	310	355	100	107	165	165	115	121	144	153	112	112
TV	115	115	113	128	213	353	0	0	159	159	138	138	138	161	112	112
TV	111	115	105	105	209	213	104	106	165	171	123	127	108	142	89	112
TV	111	111	115	115	202	215	102	104	167	167	115	125	138	155	112	114
TV	111	115	0	0	219	219	102	107	167	171	115	123	108	166	112	112
TV	115	115	103	103	202	321	98	104	165	165	115	125	138	140	112	112
TV	111	115	105	107	287	307	102	104	157	167	127	145	108	140	106	112
TV	111	119	98	98	196	360	94	100	165	165	115	123	170	170	112	112
TV	111	115	103	105	202	303	104	104	167	169	115	123	108	138	112	112
TV	111	111	96	111	202	333	102	104	157	165	117	134	108	161	112	112
TV	111	115	113	113	236	351	102	104	167	171	127	130	142	142	112	112
TV	111	115	103	115	347	349	104	107	167	169	115	123	138	140	112	112
TV	111	115	103	103	225	345	104	109	165	167	123	130	138	144	112	112
TV	115	126	269	269	202	328	100	102	158	175	115	117	134	138	110	112
TV	115	119	113	113	202	236	98	102	171	172	125	130	140	142	112	112
TV	111	119	96	111	313	319	98	100	165	171	113	123	138	138	112	112
TV	111	115	113	113	309	353	98	111	162	165	117	143	163	163	112	112
TV	111	119	103	103	200	375	100	106	167	175	121	130	138	180	112	112
TV	119	119	124	124	326	353	100	102	165	167	116	125	138	138	112	112
TV	111	115	0	0	207	375	102	102	173	173	119	119	108	144	114	114

TV	115	115	103	113	215	364	100	102	165	165	113	134	108	144	112	112
TV	111	119	103	103	209	321	100	106	171	171	121	127	142	157	112	112
TV	111	115	102	102	213	340	98	102	165	167	125	130	138	140	110	112
VL	111	115	103	103	219	317	102	104	177	177	134	136	138	146	112	112
VL	111	115	107	107	204	353	102	104	167	167	125	140	108	140	112	114
VL	111	115	103	117	340	340	98	102	165	173	121	121	140	146	112	112
VL	111	115	103	103	213	306	102	170	167	167	115	119	108	157	112	112
VL	111	119	102	102	207	332	100	102	165	171	127	132	134	142	89	112
VL	111	115	103	147	224	224	102	111	169	171	125	143	136	142	112	112
VL	111	111	128	128	225	321	102	107	169	169	123	132	108	138	112	114
VL	115	119	103	155	329	386	100	102	160	167	123	127	108	140	112	112
VL	111	115	109	109	221	360	106	107	167	171	125	134	142	153	112	112
VL	111	111	103	111	202	221	98	100	157	167	117	132	108	161	112	112
VL	115	119	103	113	324	356	102	111	165	167	121	130	140	163	112	112
VL	115	115	103	103	204	347	90	98	167	167	111	119	108	149	112	261
VL	111	126	105	122	200	219	100	100	165	167	109	130	107	142	89	112
VL	111	111	0	0	342	364	100	117	157	167	115	132	108	174	112	253
VL	111	115	105	105	299	371	102	104	165	167	113	116	140	151	112	264
VL	115	115	103	103	317	356	92	102	165	165	115	119	138	138	112	112
VL	115	123	103	103	324	390	98	107	165	175	123	132	108	140	112	112
VL	111	115	105	105	355	355	98	111	167	167	116	116	108	140	112	129
VL	111	119	109	148	209	353	102	104	157	167	115	132	146	151	112	197
VL	111	119	107	111	207	207	94	102	165	171	117	123	112	112	112	290
VL	111	111	105	105	206	332	98	107	167	171	125	130	138	140	112	112
VL	111	111	96	111	231	353	104	109	165	167	115	123	110	110	112	112
VL	115	119	103	103	314	362	106	233	165	165	123	130	138	142	112	112
VL	111	115	103	164	207	207	102	104	165	165	121	125	138	142	106	112
VL	107	126	103	113	206	334	98	100	165	165	115	136	110	138	112	112
VL	111	120	113	128	209	351	98	106	166	167	123	130	108	136	112	114
VL	115	126	134	134	196	206	102	102	165	167	113	125	110	153	106	112
SG	111	111	107	107	196	379	98	104	167	171	121	125	138	155	112	271
SG	107	111	103	103	202	211	104	106	167	167	115	115	108	182	112	112
SG	111	111	103	103	202	336	104	104	158	158	119	140	110	140	112	112
SG	111	111	103	178	311	364	98	102	165	167	119	130	140	165	112	112
LR	115	119	103	103	296	321	98	98	158	158	119	138	108	146	106	112
LR	111	111	105	105	317	355	100	172	165	165	130	150	108	180	112	112
LR	111	115	103	103	321	356	100	111	167	167	119	127	108	191	106	112
LR	115	119	103	103	217	217	107	109	167	169	119	125	138	166	112	112
LR	111	111	103	115	207	299	98	102	165	171	117	123	108	159	112	112
LR	111	126	103	103	344	370	102	104	167	171	115	115	108	161	89	89
LR	111	115	103	140	311	321	96	106	165	169	113	130	151	182	110	112
LR	111	115	103	103	223	364	102	102	173	173	117	121	142	144	112	112
LR	111	134	0	0	308	308	94	104	165	167	115	132	138	212	112	112
LR	111	119	105	105	328	358	98	104	167	167	115	125	110	144	112	112

LR	111	111	103	103	204	206	94	148	165	165	119	132	108	138	112	114
LR	111	119	107	107	344	379	100	109	165	167	123	127	108	140	112	112
LR	119	126	105	107	273	340	102	109	173	177	119	132	108	138	106	112
LR	111	115	0	0	202	223	104	104	167	167	130	132	138	138	112	112
LR	111	111	103	128	200	202	107	201	157	167	117	140	108	138	112	112
LR	111	119	107	126	199	231	94	104	157	167	119	125	138	140	112	112
LR	111	119	103	120	209	313	100	102	163	171	123	125	140	187	110	112
LR	111	115	122	122	351	356	102	102	160	167	115	117	142	144	112	114
LR	111	119	103	105	206	306	94	104	157	159	116	125	134	138	112	112
LR	111	115	103	103	317	368	102	106	160	165	111	132	108	134	0	0
LR	111	111	0	0	206	233	100	100	167	167	127	156	110	140	112	112
LR	111	112	103	103	200	231	98	100	167	169	121	121	142	144	112	112
LR	111	111	103	103	317	319	102	107	165	165	115	119	108	138	112	112
LR	115	115	103	103	194	307	96	102	165	171	127	134	138	165	112	112
LR	107	115	138	138	246	317	106	106	163	163	115	119	108	142	112	112
LR	115	119	103	103	349	364	96	142	157	171	123	125	107	146	112	112
LR	111	119	132	132	0	0	94	102	157	157	109	119	103	140	112	112
LR	111	115	105	111	0	0	102	107	165	167	121	127	140	140	112	112
LR	119	123	159	159	221	356	104	106	157	167	115	115	108	140	89	112
LR	115	119	103	164	300	328	94	109	171	171	119	123	140	157	112	112
RB	111	111	0	0	291	291	104	107	157	162	119	127	138	153	112	112
RB	111	115	0	0	200	294	92	104	165	167	115	119	108	108	112	112
RB	111	111	103	103	0	0	102	168	158	171	117	127	108	108	112	112
RB	111	115	124	124	309	324	102	104	165	167	115	119	108	142	112	112
RB	111	115	103	140	215	217	96	102	165	165	115	123	140	149	110	112
RB	111	111	103	157	318	364	100	113	158	167	115	121	108	138	0	0
RB	111	111	103	103	358	358	98	104	165	167	123	125	138	140	89	89
RB	111	126	113	113	209	366	100	104	165	171	115	115	107	153	112	255
RB	111	112	105	105	310	375	100	102	165	165	119	121	140	149	112	112
RB	115	115	103	113	283	336	96	98	165	165	115	115	108	144	89	112
RB	115	130	103	103	364	380	94	100	167	169	115	134	149	153	110	112
RB	111	115	103	122	202	383	102	102	167	171	119	127	138	140	112	112
RB	115	119	130	130	209	209	102	106	165	169	119	127	110	136	112	240
RB	111	119	105	159	375	406	94	104	167	167	125	127	108	138	112	269
RB	111	119	103	177	204	226	98	100	167	171	132	145	149	161	112	112
RB	111	111	184	184	202	240	100	106	165	167	115	125	108	108	95	280
RB	111	119	172	172	219	219	102	104	167	170	123	123	108	140	112	112
RB	111	115	103	103	209	225	102	107	165	165	121	123	142	157	112	224
RB	112	134	103	103	215	379	102	102	169	173	117	123	157	176	89	112
RB	111	111	107	178	340	356	102	106	165	167	119	119	151	165	112	112
RB	111	119	103	197	219	340	94	102	165	167	123	125	108	140	112	112
RB	111	111	103	103	202	336	102	109	169	173	125	145	151	159	112	112
RB	119	119	105	105	217	345	94	115	165	165	115	119	108	110	110	112
RB	115	119	0	0	304	355	102	104	167	167	119	127	140	151	114	114

RB	111	119	103	103	196	215	102	106	167	167	115	130	108	142	112	296
RB	111	111	103	113	206	377	100	104	171	171	134	140	108	138	112	114
RB	111	115	107	107	206	375	100	109	157	167	123	123	140	165	112	112
RB	111	115	105	201	213	287	96	106	165	166	119	125	108	166	112	112
RB	111	115	105	105	291	334	102	102	171	175	115	127	140	140	112	112
RB	111	130	103	103	211	360	102	102	165	167	119	130	108	138	112	112
RB	111	119	134	134	226	377	92	106	165	167	117	125	140	159	110	112
RB	112	115	103	103	379	379	98	104	167	167	123	138	108	155	89	112
CB	111	119	103	103	202	324	94	104	165	165	117	125	108	108	112	112
CB	111	111	103	118	225	241	170	170	158	167	127	136	138	142	112	112
CB	111	126	132	132	209	225	100	102	165	167	116	119	107	173	110	112
CB	115	115	0	0	215	342	100	102	167	167	127	136	110	172	112	112
CB	115	115	103	103	204	308	102	102	167	167	123	130	166	166	112	294
CB	111	115	103	103	234	334	92	104	167	171	119	123	108	142	112	112
CB	115	115	124	124	200	202	100	102	173	173	115	125	108	138	112	112
CB	115	119	92	92	379	379	98	109	157	167	125	125	108	142	110	112
CB	111	126	103	103	368	368	100	107	160	165	113	117	108	140	112	112
CB	111	111	250	250	322	330	104	106	167	173	115	115	142	195	0	0
CB	111	119	120	120	207	340	106	106	167	167	125	125	108	112	112	114
CB	111	115	172	172	326	353	102	102	167	172	115	121	108	138	112	112
CB	115	115	103	182	332	351	102	214	165	165	115	125	140	153	89	112
CB	111	111	0	0	204	342	102	102	167	169	115	115	108	146	89	89
CB	111	126	0	0	317	386	98	98	167	169	125	132	108	140	112	292
CB	111	111	0	0	304	304	104	104	159	171	127	127	108	168	112	112
CB	111	126	103	105	200	337	100	102	167	167	119	123	108	140	112	112
CB	111	111	103	103	216	221	100	106	167	167	109	127	142	186	112	112
CB	111	126	128	128	0	0	96	111	165	171	119	119	151	165	106	303
CB	111	119	103	103	207	353	102	199	167	171	123	125	153	155	112	112
CB	111	134	178	178	204	340	100	104	170	170	121	127	138	165	112	229
CB	107	111	103	103	204	336	98	157	158	158	115	123	108	153	112	112
CB	111	119	128	138	206	221	104	113	165	167	115	119	142	197	110	110
CB	111	115	103	103	209	386	102	109	165	175	119	127	110	110	112	114
CB	111	119	0	0	294	347	98	102	165	167	113	130	140	146	112	278
CB	115	126	103	134	353	353	98	106	158	171	123	123	111	136	89	112
CB	111	126	155	155	237	347	98	102	167	167	121	132	138	138	112	200
CB	111	119	105	105	317	345	107	109	160	171	115	123	140	155	0	0
CB	111	111	103	136	202	336	90	102	157	167	113	115	138	144	112	112
SB	111	115	103	103	211	232	96	102	158	167	115	125	110	140	112	112
SB	111	111	0	0	352	383	96	104	162	165	119	121	140	157	112	222
SB	111	111	136	136	236	356	102	170	163	167	119	130	138	217	110	112
SB	107	111	103	103	321	321	100	104	165	167	121	132	108	146	112	112
SB	111	111	103	103	353	366	98	106	157	165	119	119	136	182	110	112
SB	111	111	109	109	215	217	98	104	167	167	119	134	110	138	112	112
SB	111	115	105	134	340	340	98	106	165	177	115	123	108	140	112	112

SB	111	111	103	103	223	336	102	102	167	167	113	119	138	140	112	112
SB	111	111	103	103	356	383	104	107	165	165	117	127	140	180	112	112
SB	111	111	128	128	207	231	96	104	165	173	115	121	108	140	112	112
SB	111	115	111	132	207	401	98	107	171	171	115	127	107	142	112	112
SB	111	115	0	0	317	326	106	210	171	173	117	143	134	157	112	112
SB	111	115	134	134	206	206	98	100	160	160	123	125	108	155	112	112
SB	111	115	0	0	330	356	98	107	165	165	115	115	108	151	112	112
SB	115	115	103	126	192	217	102	109	165	167	115	117	144	146	112	112
SB	111	119	98	147	355	355	94	109	157	165	127	134	108	140	112	112
SB	111	111	103	103	206	219	100	104	165	167	125	125	136	155	112	112
SB	111	115	140	140	207	338	106	109	158	165	127	127	142	146	116	197
SB	115	115	113	126	330	330	104	206	167	167	119	132	108	144	112	303
SB	108	115	103	103	302	321	98	100	167	171	115	140	140	140	112	112
SB	111	126	208	208	221	303	98	100	165	165	117	119	140	163	95	95
SB	111	123	105	113	206	342	100	102	165	167	115	117	142	161	112	112
SB	111	111	103	103	206	207	100	102	165	167	119	119	140	151	112	114
SB	115	115	103	117	329	336	98	109	165	167	136	140	108	144	112	112
SB	119	119	0	0	200	334	102	104	157	169	119	119	136	140	112	112
SB	111	115	103	122	213	213	100	210	157	165	115	134	144	153	112	114
SB	111	111	0	0	206	206	102	170	165	169	115	136	108	108	112	112
SB	111	111	103	105	231	311	92	106	157	167	115	121	136	153	112	112
SB	111	115	103	103	313	368	107	182	167	167	115	116	142	174	112	112
SB	111	119	103	103	349	349	100	100	163	165	117	119	108	140	112	112
SB	115	119	103	103	246	410	92	92	158	167	119	132	138	140	112	112
SB	111	119	117	128	304	355	96	106	165	167	125	134	151	159	112	112
SB	111	115	0	0	202	225	98	102	165	165	115	130	108	149	112	137
EB	111	111	103	103	209	213	98	104	165	167	119	119	136	142	112	114
EB	111	111	103	103	0	0	102	106	167	169	119	132	110	142	106	112
EB	111	123	103	103	206	219	102	102	165	168	119	132	142	151	112	112
EB	111	120	113	130	0	0	102	106	165	167	119	130	136	144	112	112
EB	107	111	126	126	204	209	98	106	160	169	123	134	155	176	112	112
EB	111	115	103	103	310	390	100	102	171	171	115	123	111	142	112	112
EB	107	119	102	103	344	351	94	100	165	167	119	119	138	140	112	112
EB	111	119	0	0	207	309	98	100	169	171	115	125	108	140	112	135
EB	111	111	105	142	217	375	104	109	158	167	119	121	108	140	110	112
EB	111	126	103	105	206	379	98	104	167	169	115	127	108	142	110	112
EB	111	111	118	126	200	319	102	117	165	167	113	119	110	174	112	112
EB	111	111	103	103	386	386	94	313	157	165	125	132	108	182	112	112
EB	111	119	105	105	200	287	96	98	167	167	115	119	108	126	112	112
EB	111	119	120	128	336	336	98	102	165	165	123	132	108	140	112	259
EB	111	111	103	103	207	379	102	195	157	165	119	123	108	113	112	265
EB	115	115	100	100	295	368	98	142	157	157	119	119	140	140	112	112
EB	111	111	103	166	213	334	94	102	167	171	119	125	155	163	112	155
EB	111	115	0	0	206	330	102	104	157	169	115	134	138	184	112	112

EB	115	115	103	103	321	338	96	98	165	167	119	125	138	182	0	0
EB	115	126	113	126	336	338	102	106	165	165	119	125	108	138	112	112
EB	111	111	120	120	371	371	102	104	157	167	123	136	140	151	112	112
EB	111	115	0	0	235	375	98	102	165	167	115	136	108	140	112	112
EB	115	119	98	103	297	317	100	102	167	167	119	119	108	108	112	424
EB	111	119	0	0	204	213	102	106	165	169	115	130	108	108	110	112
EB	111	115	103	103	206	206	102	104	167	167	115	132	108	157	95	95
EB	115	115	103	128	226	342	98	107	167	171	132	134	108	144	112	114
EB	111	111	124	124	336	375	106	107	167	167	117	119	149	159	112	112
EB	111	115	103	103	192	375	98	102	169	169	117	136	161	163	112	112
EB	107	119	103	103	213	319	111	111	157	169	119	119	144	186	110	112
EB	111	111	122	122	206	307	104	104	167	173	125	127	140	140	89	112
EB	115	115	103	103	234	349	106	107	165	167	121	121	108	142	112	112
EB	115	123	235	235	314	349	100	109	158	165	117	132	110	113	112	112
EB	111	115	117	117	347	356	100	107	165	167	116	125	108	140	112	112
EB	115	115	103	103	219	219	106	109	165	165	115	125	108	153	112	112
ES	111	115	113	172	200	273	100	104	167	167	130	130	108	110	112	114
ES	111	115	103	103	202	329	98	98	165	167	116	130	108	191	112	112
ES	115	119	103	103	204	360	102	102	167	170	121	138	108	178	112	112
ES	111	115	100	105	373	388	98	98	165	173	115	127	110	149	112	112
ES	115	126	103	178	217	217	102	216	165	165	115	115	140	142	112	112
ES	111	111	103	103	202	326	104	107	165	165	119	125	108	159	112	112
ES	115	115	103	113	379	379	100	107	162	165	130	132	108	168	112	237
ES	119	126	105	115	330	356	104	189	165	167	115	123	108	140	112	131
ES	115	115	136	159	207	344	104	106	167	167	121	123	108	140	0	0
ES	111	115	103	103	200	206	98	104	167	167	109	115	146	146	112	112
ES	115	123	103	235	244	340	100	107	165	171	119	134	108	163	112	112
ES	111	111	103	103	204	285	102	102	165	165	123	125	110	146	110	112
ES	111	119	103	103	279	383	98	100	157	170	119	125	140	140	112	114
ES	111	111	103	103	206	207	102	102	167	169	119	123	168	193	112	139
ES	111	111	103	161	206	209	92	104	165	171	115	121	110	149	112	112
ES	111	126	103	103	237	324	102	159	167	167	109	119	108	140	112	114
ES	115	126	103	103	231	317	104	107	167	167	115	125	108	110	112	112
ES	111	115	103	107	206	360	100	176	165	167	123	130	138	142	112	112
ES	115	119	102	102	307	347	102	106	162	165	115	127	108	146	112	112
ES	111	111	105	105	212	311	102	160	165	167	115	134	140	176	112	112
ES	111	115	103	103	221	291	98	100	157	157	111	140	138	138	112	112
ES	111	111	107	107	219	309	102	104	165	167	117	121	108	149	112	112
ES	119	119	0	0	204	287	92	102	165	167	127	130	138	155	112	112
ES	111	111	109	164	0	0	102	107	165	165	121	130	111	140	112	114
ES	107	115	98	153	281	360	92	107	165	171	115	119	110	142	112	112
ES	107	115	142	142	0	0	107	109	157	169	117	125	108	159	112	201
ES	111	111	107	107	219	309	102	104	165	167	117	121	108	149	112	112
ES	111	111	103	105	190	299	100	100	167	167	116	130	108	155	112	112

ES	115	115	126	174	321	326	109	109	165	165	113	121	108	140	112	112
ES	111	126	124	124	315	319	92	102	165	167	121	123	140	140	0	0
IM	115	119	103	103	237	241	98	102	157	165	115	123	108	108	112	112
IM	111	111	128	128	229	375	94	106	165	167	115	123	110	140	110	112
IM	111	111	103	103	206	231	100	104	167	171	121	123	108	108	112	112
IM	111	115	113	113	192	202	100	104	165	167	140	143	138	140	112	135
IM	111	111	0	0	221	340	92	102	167	167	119	138	108	138	112	112
IM	115	115	103	103	202	206	102	106	165	165	119	127	140	149	112	112
IM	111	126	103	115	202	345	102	102	171	171	119	127	108	142	114	247
IM	115	115	103	107	202	206	102	106	165	165	119	127	140	149	112	112
IM	111	115	128	130	209	221	100	281	165	167	117	119	108	136	0	0
IM	111	126	103	130	222	332	102	104	165	165	115	127	108	165	112	112
IM	111	119	103	138	321	321	94	100	165	169	125	136	138	140	112	230
IM	111	111	103	103	209	215	102	106	165	171	115	116	140	146	112	112
IM	111	115	103	103	202	221	94	102	167	167	113	119	108	142	112	112
IM	119	123	142	142	328	330	94	100	169	171	125	132	142	163	112	112
IM	115	115	103	208	192	208	100	102	167	171	130	132	108	165	0	0
IM	111	111	103	103	340	340	100	102	165	165	119	132	108	136	112	112
IM	111	115	166	166	217	330	102	113	165	171	115	125	142	189	112	112
IM	111	119	103	103	200	326	104	109	171	171	119	119	108	140	112	112
IM	111	111	107	109	330	330	100	102	165	165	127	132	108	108	112	135
IM	111	126	103	128	246	334	96	98	157	165	119	119	138	140	112	112
IM	111	119	105	126	206	215	100	102	157	167	125	161	140	142	112	112
IM	111	115	120	122	223	326	104	106	167	171	115	130	108	108	112	112
IM	111	119	103	124	312	334	96	102	165	165	116	119	108	140	112	112
IM	111	115	103	103	291	347	98	102	157	165	115	134	144	186	112	112
IM	111	115	113	113	209	326	102	107	167	173	121	125	155	170	112	112
IM	115	119	105	105	360	375	102	140	157	171	121	127	138	140	112	112
IM	111	111	126	126	209	246	98	219	165	167	119	123	138	142	112	112
IM	111	111	103	178	207	353	98	102	165	168	115	117	108	140	112	112
IM	111	111	103	103	328	328	100	102	167	171	130	132	108	155	112	255
IM	111	111	105	170	202	202	104	106	165	169	119	138	110	142	112	294
ML	111	111	0	0	330	342	102	109	167	171	119	119	138	138	112	112
ML	115	119	103	103	317	347	98	100	165	167	109	127	142	168	112	112
ML	115	126	103	107	237	237	106	109	167	167	119	123	112	140	112	112
ML	111	111	126	126	192	206	96	142	155	165	132	150	140	144	112	112
ML	115	119	103	103	344	355	102	104	165	167	115	130	140	166	112	131
ML	111	111	102	103	377	377	92	100	157	167	117	136	108	138	112	112
ML	111	115	0	0	219	356	100	104	157	165	109	115	108	174	112	112
ML	111	119	103	103	231	362	98	102	171	171	119	130	140	153	112	112
ML	111	111	103	103	229	362	96	102	165	167	119	130	108	138	112	112
ML	111	115	126	126	202	303	94	104	165	165	127	130	138	142	110	112
ML	111	115	103	103	207	375	94	106	165	165	119	136	112	144	89	89
ML	111	111	103	103	207	221	94	102	165	165	134	138	142	142	112	294

ML	111	111	120	122	349	349	94	100	165	167	123	123	108	138	112	112
ML	111	115	103	103	287	326	96	106	165	171	134	143	138	149	0	0
ML	111	115	103	103	221	307	92	106	165	167	130	138	108	186	89	112
ML	115	115	113	113	225	229	98	106	165	167	123	125	140	144	112	112
ML	111	115	98	98	202	349	98	107	165	169	113	123	138	146	112	112
ML	111	119	164	164	194	306	104	164	165	169	121	123	110	112	112	112
ML	111	115	103	105	215	309	102	104	165	165	119	121	108	180	112	112
ML	111	115	208	208	204	351	106	111	165	167	119	119	108	166	112	237
ML	111	119	105	105	200	319	106	107	165	165	119	134	138	153	112	112
ML	111	119	103	103	207	345	100	104	165	167	117	123	140	163	112	112
ML	111	126	126	126	345	383	100	104	157	167	115	127	138	149	112	114
ML	111	115	103	126	232	279	102	106	165	167	115	134	101	138	112	135
ML	107	111	193	193	353	383	96	102	173	173	134	136	138	140	89	112
ML	115	119	151	151	230	237	98	203	177	177	115	117	142	172	112	112
ML	111	115	102	102	198	217	102	102	167	171	115	121	108	108	112	112
ML	115	119	98	98	206	213	102	106	165	171	119	119	108	140	112	112
ML	111	115	103	103	206	315	94	106	167	172	115	125	138	252	112	112
ML	111	111	103	103	202	219	98	102	163	171	123	125	108	138	112	112
TT	111	115	103	103	223	226	100	102	157	173	123	140	108	155	112	112
TT	111	119	159	159	207	386	102	104	167	167	119	119	108	140	112	116
TT	111	115	126	126	315	315	98	109	165	165	115	119	138	146	112	220
TT	115	119	105	151	330	330	96	106	165	167	127	127	134	151	112	273
TT	111	111	105	105	202	207	102	102	167	167	121	130	140	146	112	131
TT	115	115	103	103	230	313	100	102	159	167	127	152	138	138	112	112
TT	115	115	103	103	215	330	100	106	165	173	115	119	138	138	112	112
TT	111	111	103	157	221	336	102	111	167	168	117	130	108	142	112	112
TT	115	119	103	140	215	221	102	104	165	171	113	125	132	138	112	112
TT	115	119	103	103	211	220	94	100	157	165	115	134	146	186	112	112
TT	111	111	98	98	289	289	106	109	162	167	125	130	108	138	112	112
TT	111	115	118	126	351	386	98	104	165	165	127	136	110	138	112	237
TT	111	115	105	105	221	364	102	104	157	167	115	119	108	138	112	239
TT	115	126	0	0	229	366	172	191	165	167	115	119	108	170	112	282
TT	111	115	103	105	213	309	100	104	166	167	119	119	138	140	112	112
TT	111	115	105	105	190	308	102	104	165	171	113	115	108	178	112	112
TT	111	119	103	118	292	356	102	106	167	167	115	119	108	140	112	208
TT	111	111	103	103	206	206	102	106	165	167	115	132	138	138	112	112
TT	111	111	103	113	202	226	94	109	165	167	119	127	163	184	112	112
TT	111	126	103	103	231	336	98	102	167	169	115	117	108	108	112	112
TT	111	115	103	201	206	211	96	102	157	160	119	145	108	138	112	112
TT	111	111	103	115	321	324	102	106	165	171	119	127	149	151	110	112
TT	111	111	103	130	215	215	100	102	165	167	119	145	108	155	112	112
TT	111	119	98	98	204	356	98	102	158	177	115	123	142	170	112	112
TT	115	115	98	103	332	340	102	109	165	171	127	130	108	140	112	131
TT	111	119	113	113	255	326	102	109	165	171	123	125	108	166	112	112

TT	111	111	113	113	351	425	94	104	167	167	121	130	140	159	112	112
TT	111	115	103	103	198	236	100	109	171	175	115	117	108	140	112	112
TT	115	119	189	189	326	379	102	106	157	169	123	132	140	146	112	112
TT	111	119	103	103	230	281	100	104	166	167	125	134	138	144	112	112
BJ	115	115	105	105	213	362	96	96	165	165	127	130	142	182	112	112
BJ	115	115	142	142	226	371	102	102	167	167	121	130	140	151	112	112
BJ	107	111	105	136	336	340	106	109	167	167	115	132	107	138	110	112
BJ	111	115	0	0	200	207	104	107	165	165	115	115	108	138	89	112
BJ	111	119	0	0	202	209	98	100	165	167	115	127	108	108	112	112
BJ	115	126	105	105	333	360	98	100	163	163	115	145	138	157	89	112
BJ	111	115	124	124	296	296	106	107	157	157	115	119	108	182	112	112
BJ	111	119	103	159	200	321	96	102	157	157	115	119	108	108	114	259
BJ	111	111	178	195	356	375	102	172	165	165	119	125	108	236	112	112
BJ	111	115	113	113	202	342	100	102	165	165	115	121	140	140	112	265
BJ	115	115	103	120	330	334	92	96	157	165	115	125	108	155	112	112
BJ	111	115	103	103	356	356	98	100	165	167	119	123	110	144	112	112
BJ	111	111	111	136	355	355	100	102	167	171	0	0	142	189	112	112
BJ	111	111	103	117	304	338	104	109	165	167	113	125	108	142	112	112
BJ	111	115	124	222	313	390	104	104	0	0	127	127	140	146	0	0
BJ	115	115	103	105	309	317	98	102	163	171	115	123	163	172	112	249
BJ	111	119	103	103	188	340	94	104	157	171	123	125	108	144	0	0
BJ	111	112	105	138	207	287	96	102	157	165	115	130	108	138	112	112
BJ	115	115	0	0	217	219	100	100	167	167	117	130	108	134	95	112
BJ	111	115	103	103	309	309	98	102	165	167	115	115	108	171	112	112
BJ	111	115	115	120	200	217	92	96	165	171	115	130	110	157	112	112
BJ	111	115	193	193	206	364	96	109	158	165	117	125	108	140	112	114
BJ	111	115	120	120	190	340	102	106	165	167	109	121	110	140	112	118
BJ	111	111	0	0	200	207	104	201	155	157	125	130	110	187	112	112
BJ	115	119	98	98	200	200	100	102	165	167	127	132	138	155	112	112
BJ	111	119	103	107	355	355	98	106	165	175	115	134	142	153	112	112
BJ	115	115	118	193	342	342	98	100	167	175	119	143	140	161	112	114
BJ	111	115	105	105	289	289	102	102	165	168	115	127	108	110	110	112
BJ	111	119	103	103	315	356	104	106	159	165	115	132	108	151	112	116
BJ	115	115	105	118	202	329	100	106	165	167	119	121	108	144	112	114
LB	115	115	103	103	321	353	92	104	158	171	123	125	163	165	112	112
LB	111	111	105	105	209	334	102	109	165	167	115	125	108	110	112	112
LB	115	119	103	103	358	358	100	100	167	167	119	125	138	138	112	112
LB	111	115	105	113	200	206	100	102	165	173	119	127	155	174	112	233
LB	111	111	0	0	209	362	96	98	167	171	127	127	108	144	0	0
LB	115	115	109	109	206	206	98	100	165	167	115	130	108	108	112	112
LB	111	115	98	111	230	375	102	102	167	167	119	134	140	142	110	112
LB	111	119	103	103	200	383	98	104	165	165	115	121	138	142	0	0
LB	111	126	103	122	204	328	98	104	167	169	113	123	108	140	112	112
LB	111	115	103	103	200	207	98	106	167	167	115	119	140	153	112	114

LB	111	111	103	103	206	364	98	102	157	165	117	117	108	142	112	112
LB	115	115	98	103	296	355	102	107	165	167	121	127	108	108	89	112
LB	111	119	107	122	287	351	187	210	179	179	119	127	140	140	118	118
LB	115	126	102	195	321	321	102	102	165	167	119	132	108	138	112	112
LB	111	119	113	113	313	386	102	109	163	165	125	132	108	149	112	114
LB	115	126	103	161	283	353	98	106	165	167	115	115	110	110	112	112
LB	111	115	103	151	226	371	102	109	170	171	115	132	110	214	112	112
LB	111	111	109	109	200	206	104	187	167	173	123	125	136	142	112	112
LB	115	115	103	103	188	291	102	109	158	170	123	125	140	146	0	0
LB	111	126	117	117	217	340	102	102	171	171	113	123	140	142	0	0
LB	119	126	105	105	221	307	102	107	167	167	123	134	138	144	112	112
LB	115	119	103	103	209	330	100	107	177	177	119	125	108	138	112	208
LB	111	119	98	134	200	317	102	206	166	167	119	123	108	132	112	112
LB	115	126	105	105	198	260	100	106	167	171	130	132	108	140	110	112
LB	111	119	105	105	207	221	102	104	165	175	119	121	108	168	112	135
LB	111	111	103	103	213	344	100	144	157	167	123	140	140	163	112	112
LB	111	111	103	103	204	217	98	109	165	167	119	125	108	146	112	112
LB	111	115	113	113	200	379	100	102	165	165	115	125	107	138	112	112
LB	111	115	113	113	200	379	100	102	165	165	115	125	107	138	112	112
LB	111	111	128	128	202	356	98	102	171	185	119	130	138	153	112	112
DK	111	111	103	103	206	206	102	104	165	165	115	119	110	174	110	112
DK	111	111	103	103	299	342	102	102	167	167	136	145	110	140	112	269
DK	111	115	103	103	207	353	102	102	165	165	115	130	108	108	112	112
DK	111	115	103	103	207	353	102	102	165	165	115	130	108	108	112	112
DK	111	111	103	103	206	221	96	96	167	167	119	123	108	110	112	212
DK	111	126	128	166	203	219	102	102	167	171	125	136	108	110	106	112
DK	111	111	103	103	217	287	98	109	165	173	119	119	138	153	112	112
DK	111	111	0	0	190	211	106	132	157	169	119	125	108	155	112	112
DK	111	111	138	138	215	416	98	203	170	171	121	127	138	153	112	112
DK	111	111	0	0	311	311	92	104	165	165	117	132	149	151	89	112
DK	115	119	109	109	202	353	98	101	167	167	115	115	144	172	112	112
DK	111	115	118	118	215	375	102	104	167	171	119	119	108	108	112	112
DK	111	115	98	102	206	345	98	104	165	167	119	134	108	155	89	112
DK	111	115	103	103	231	344	104	106	160	167	119	127	108	138	112	112
DK	111	115	113	113	204	315	96	102	167	167	113	123	138	142	112	112
DK	111	111	103	128	204	340	102	107	167	171	115	121	108	146	112	112
DK	111	126	103	103	233	336	100	104	165	169	115	121	108	140	112	112
DK	111	115	105	107	313	315	92	102	167	169	113	123	108	138	112	112
DK	115	115	103	103	204	349	102	106	167	167	123	127	108	138	112	112
DK	115	119	120	120	321	358	100	104	165	165	115	125	138	144	112	249
DK	107	111	103	103	204	317	104	104	167	167	119	123	140	163	112	237
DK	111	111	103	115	304	364	102	102	167	177	115	115	138	174	112	112
DK	111	119	103	103	231	410	98	109	165	165	115	115	138	149	112	112
DK	111	112	103	122	318	326	102	102	158	165	130	148	108	108	112	114

DK	111	119	92	92	237	347	106	107	157	165	117	125	108	157	112	112
DK	115	119	103	105	213	336	102	102	157	167	115	119	140	151	89	112
DK	115	119	103	103	213	336	102	102	157	167	115	119	140	151	89	112
DK	111	115	0	0	206	360	104	182	163	167	116	123	108	146	112	112
DK	115	126	103	124	236	347	102	106	165	165	115	132	138	142	112	112
DK	111	119	126	126	206	206	94	102	167	169	115	118	108	146	112	112

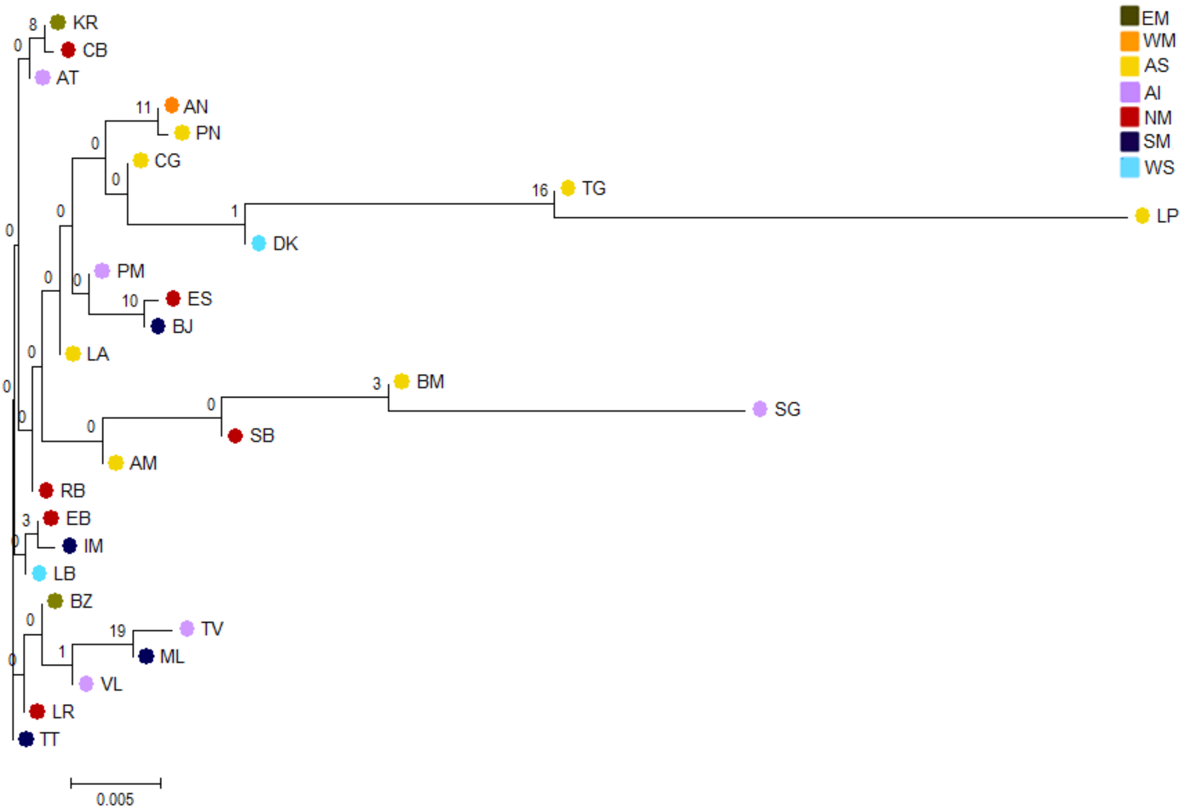

Figure S1 – Unrooted neighbour-joining tree of the pairwise genetic distance between populations of *P. perna* across northeastern Atlantic and Mediterranean shores based on proportion of shared alleles of 7 microsatellite loci, running 999 bootstraps. Colours depict distinct expected oceanographic regions. Numbers above branches correspond to bootstrap support. EM, Eastern Mediterranean; WM, Western Mediterranean; AS, Alboran Sea; AI, Atlantic Iberia; NM, northwestern Morocco; SM, Southern Morocco; WS, Western Sahara. Locations codes as in Table S6.

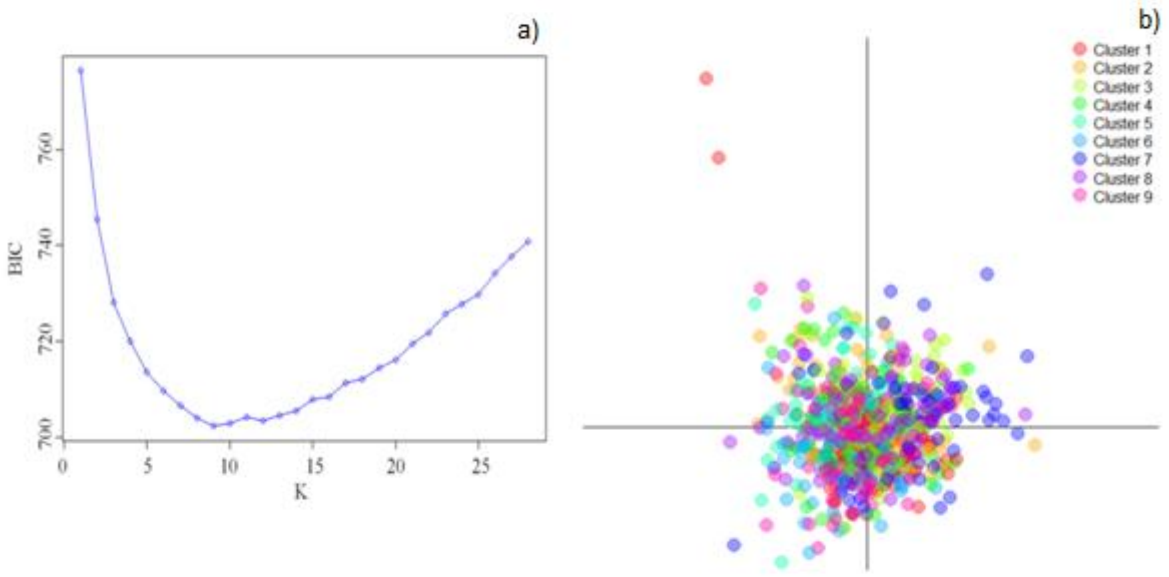

Figure S2 – Discriminant analyses of principal components (DAPC) results. a) Selection of best fitting number of clusters, K (K=9) based on Bayesian Information Criterion (BIC), b) DAPC spatial plot of the 27 populations distributed through nine clusters. Colours correspond to distinct clusters. Each individual was assigned to a cluster and solid dots correspond to the overlapping of several individuals.

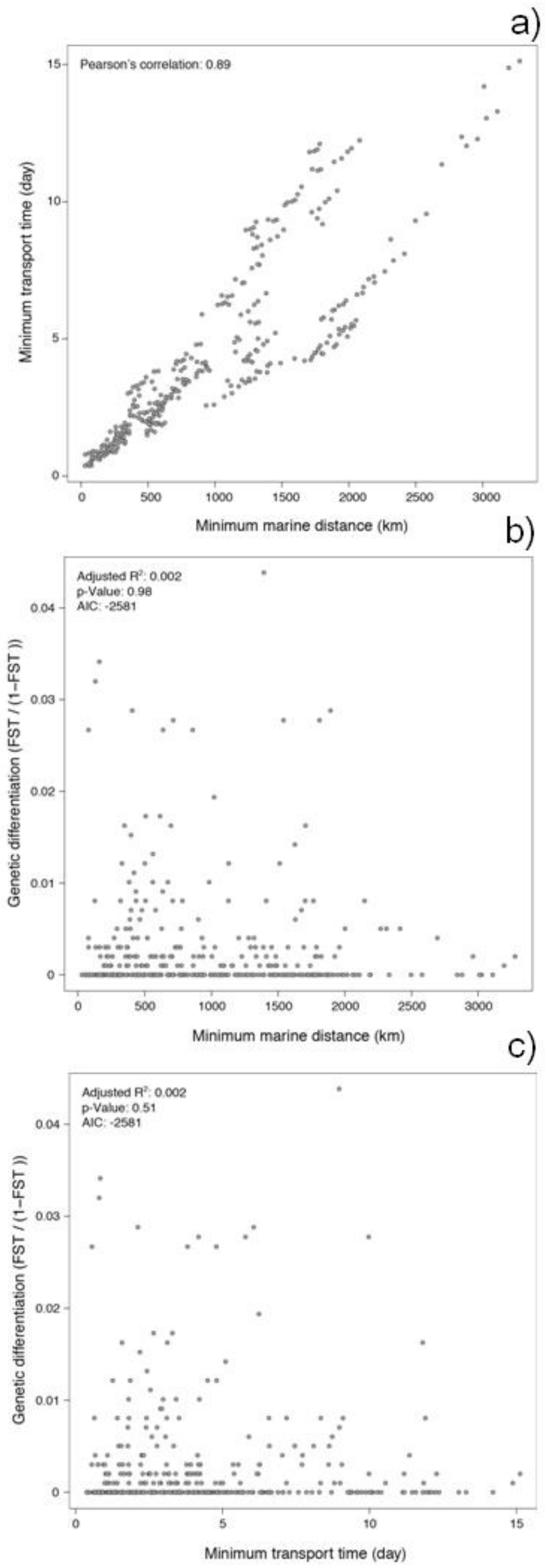

Figure S3 – Dispersal simulation of *P. perna* larvae. a) Correlation between minimum transport time (day) and minimum marine distance (km); Correlation between genetic differentiation and b) minimum marine distance (km) and c) minimum transport time (days).

References

1 Boudoresque, C. F., J.G., H. & de Grissac, J. Le benthos marin de l'Ile de z*embra (Parc National, Tunisie)*. (GIS Posidonie, 1986).

2 Belabed, B. E. *et al.* Factors contributing to heavy metal accumulation in sediments and in the intertidal mussel *Perna perna* in the Gulf of Annaba (Algeria). *Mar. Pollut. Bull.* **74**, 477-489, doi:10.1016/j.marpolbul.2013.06.004 (2013).

3 Abada-Boudjema, Y.-M. & Davin, J.-C. Recruitment and life span of two natural mussel populations *Perna perna* (Linnaeus) and *Mytilus galloprovincialis* (Lamarck) from the Algerian coast. *J. Molluscan Stud.* **61**, 467-481 (1995).

4 Klouche, M. S., De Deurwaerdère, P., Dellu-Hagedorn, F., Lakhdar-Ghazal, N. & Benomar, S. Monoamine content during the reproductive cycle of *Perna perna* depends on site of origin on the Atlantic Coast of Morocco. *Sci. Rep.* **5**, 13715, doi:10.1038/srep13715 (2015).

5 Jourmi, L. E. *et al.* Assessment of water quality in coastal environments of mohammedia applying responses of biochemical biomarkers in the brown mussel *Perna perna*. *IJCSI Int. J. Comp. Sci.* **9**, 505-510 (2012).

6 Mhamada, M., Cheihk, M. O. M., Khannous, S., Dartige, A. & Hassan, E.-R. Metallic contamination assessment of the Lévrier Bay (Mauritanian Atlantic coast), using *Perna perna* and *Venus rosalina*. *J. Env. Sol.* **3**, 1-10 (2015).

7 Roméo, M., Sidoumou, Z. & Gnassia-Barelli, M. Heavy metals in various molluscs from the Mauritanian coast. *Bull. Environ. Contam. Toxicol.* **65**, 269-276, doi:10.1007/s001280000124 (2000).

8 Sidoumou, Z., Gnassia-Barelli, M., Siau, Y., Morton, V. & Romeo, M. Heavy metal concentrations in molluscs from the Senegal coast. *Environ. Int.* **32**, 384-387, doi:10.1016/j.envint.2005.09.001 (2006).

9 Zabi, S. G. Répartition et abondance des espèces de la macrofaune benthique de la lagune Ebrié (Côte d'Ivoire). *Document Scientifique du Centre de Recherche Océanographique, Abidjan* **13** (1982).

10 Otchere, F. A., Joiris, C. R. & Holsbeek, L. Mercury in the bivalves *Anadara* (*Senilia*) *senilis*, *Perna perna* and *Crassostrea tulipa* from Ghana. *Sci. Total Environ.* **304**, 369-375, doi:10.1016/s0048-9697(02)00582-x (2003).

11 Cayré, P. Étude de la moule *Perna perna* L. et des possibilities de Myticulture en République Populaire du Congo. *Cahiers ORSTOM. Serie Oceanographie* **16**, 9-17 (1978).

12 Sokołowski, A., Bawazir, A. S., Sokołowska, E. & Wołowicz, M. Seasonal variation in the reproductive activity, physiological condition and biochemical components of the brown mussel *Perna perna* from the coastal waters of Yemen (Gulf of Aden). *Aquat. Living Resour.* **23**, 177-186, doi:10.1051/alr/2010016 (2010).

13 Szefer, P. & Geldon, J. Distribution and association of trace metals in soft tissue and byssus of mollusc *Perna perna* from the Gulf of Aden, Yemen. *Environ. Int.* **23**, 53-61 (1997).

14 Badawy, M. I. & Al-Harthy, F. Hydrocarbons in seawater, sediment, and oyster from the Omani coastal waters. *Bull. Environ. Contam. Toxicol.* **47**, 386-391 (1991).

15 Cunha, R. L. *et al.* Wider sampling reveals a non-sister relationship for geographically contiguous lineages of a marine mussel. *Ecol. Evol.* **4**, 2070-2081, doi:10.1002/ece3.1033 (2014).
